# Characterization and Validation of Arg286 Residue of IL-1RAcP as a Potential Drug Target for Osteoarthritis

**DOI:** 10.3389/fchem.2020.601477

**Published:** 2021-02-03

**Authors:** Angela Dailing, Kelsey Mitchell, Ngoc Vuong, Kyung Hyeon Lee, Reva Joshi, Virginia Espina, Amanda Haymond Still, Carter J. Gottschalk, Anne M. Brown, Mikell Paige, Lance A. Liotta, Alessandra Luchini

**Affiliations:** ^1^Center for Applied Proteomics and Molecular Medicine, Institute for Advanced Biomedical Research, George Mason University, Manassas, VA, United States; ^2^Department of Chemistry and Biochemistry, Institute for Advanced Biomedical Research, George Mason University, Manassas, VA, United States; ^3^Department of Biochemistry, Virginia Tech, Blacksburg, VA, United States; ^4^Research and Informatics, University Libraries Virginia Tech, Blacksburg, VA, United States

**Keywords:** molecular dynamics, molecular modeling, protein painting, peptide, targeted inhibitors

## Abstract

Osteoarthritis (OA) is the most common form of arthritis and the fastest growing cause of chronic disability in the world. Formation of the ternary IL-1β /IL-1R1/IL-1RAcP protein complex and its downstream signaling has been implicated in osteoarthritis pathology. Current OA therapeutic approaches target either the cytokine IL-1β or the primary receptor IL-1RI but do not exploit the potential of the secondary receptor IL-1RAcP. Our previous work implicated the Arg286 residue of IL-1RAcP as a key mediator of complex formation. Molecular modeling confirmed Arg286 as a high-energy mediator of the ternary IL-1β complex architecture and interaction network. Anti-IL-1RAcP monoclonal antibodies (mAb) targeting the Arg286 residue were created and were shown to effectively reduce the influx of inflammatory cells to damaged joints in a mouse model of osteoarthritis. Inhibitory peptides based on the native sequence of IL-1RAcP were prepared and examined for efficacy at disrupting the complex formation. The most potent peptide inhibitor had an IC_50_ value of 304 pM in a pull-down model of complex formation, and reduced IL-1β signaling in a cell model by 90% at 2 μM. Overall, therapies that target the Arg286 region surface of IL-1RAcP, and disrupt subsequent interactions with subunits, have the potential to serve as next generation treatments for osteoarthritis.

## Introduction

Osteoarthritis (OA), the most common form of arthritis, is a severely debilitating disease causing suffering for more than 300 million people worldwide (Brown et al., 2006; Kloppenburg and Berenbaum, 2020). OA is the fastest growing cause of chronic disability in the world, and its incidence is expected to increase with extended life expectancy and aging of the global population (Murray et al., 2012; Safiri et al., 2020). The global cost of OA, including medical care expenditures and lost work productivity, has been estimated to be 0.25–1.1% of a country gross domestic product (GDP) (Puig-Junoy and Ruiz Zamora, 2015; Zhao et al., 2019). OA is characterized by degenerative changes in articular cartilage, bone, and associated joint tissues. Post-traumatic arthritis (PTA), one of the etiologic subtypes of osteoarthritis, is a leading cause of joint disability and it is thought to represent 12% of all OA cases (Punzi et al., 2016). PTA can follow a wide variety of joint or joint-associated tissue injuries including ligament and meniscal tears and articular impact (Roos et al., 1998; Lohmander et al., 2007; Chu et al., 2010). Articular fractures commonly cause accelerated joint degeneration (Furman et al., 2006). Surgical reduction and fixation of the joint does not prevent the development of PTA. Therefore, there is an urgent need to develop novel therapeutic approaches for PTA after joint injury.

**Figure 1:**
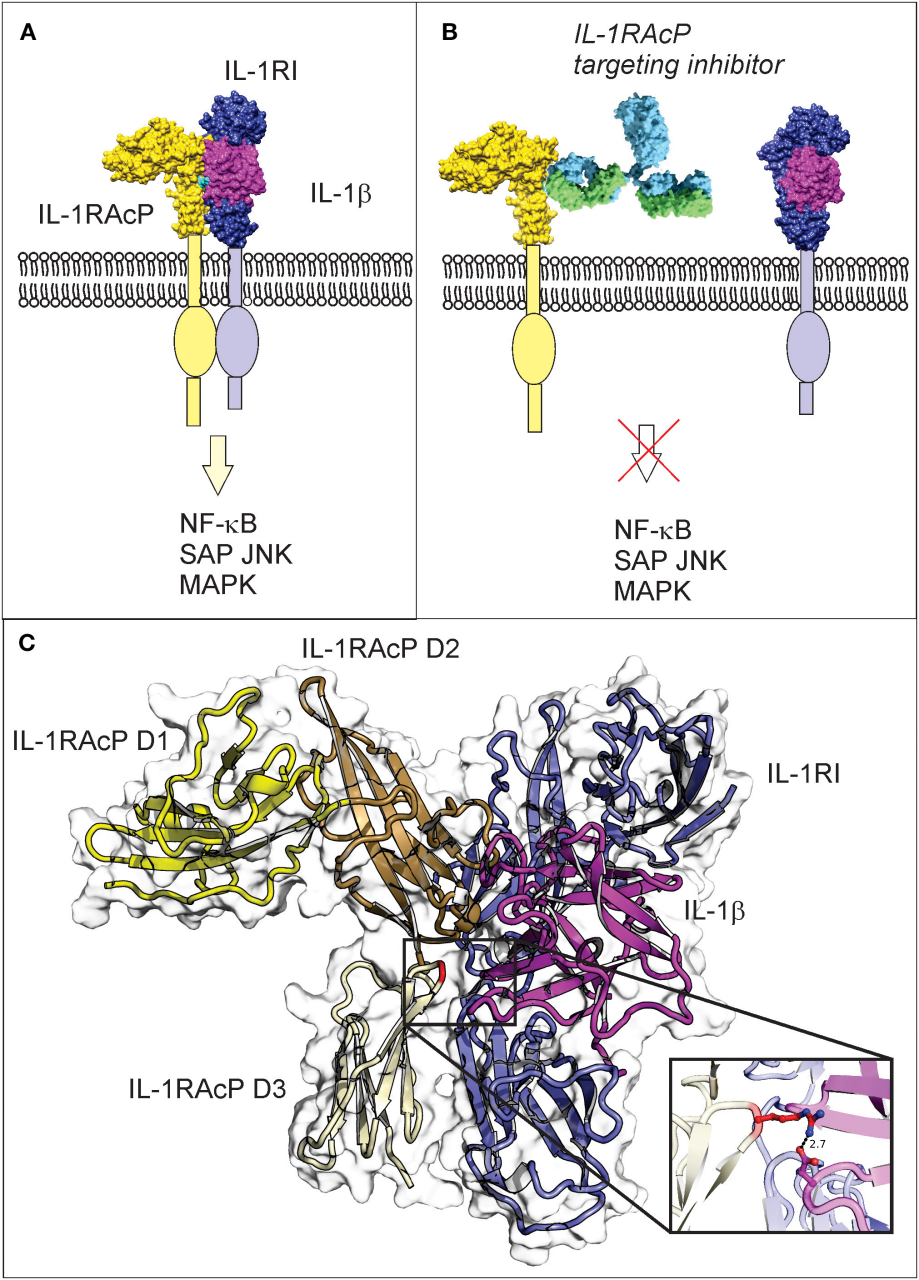
IL-1β three-way complex formation with IL-1R1 and IL-1RAcP is required for inflammatory signaling, with the Arg286 residue of IL-1RAcP found at the three-way interface. **(A)** Engagement of IL-1RAcP with the pre-formed receptor-ligand complex triggers downstream signaling. **(B)** An inhibitory peptide that prevents the three-way complex formation will inhibit SAPK/JNK signaling downstream from IL-1RI:IL-1RAcP. **(C)** The crystal structure of the ternary IL-1β complex shows the three-way interaction of IL-1RI (shown in blue), IL-1β (shown in magenta), and IL-1RAcP (shown in yellow). IL-1RAcP is composed of three immunoglobulin-like domains (D1–D3) highlighted in the figure. The Arg286 hot spot residue is highlighted in red and displayed in the close-up view. Chimera 1.11.12 was used to view the crystal structure using PBD ID 4dep.

Current osteoarthritis drug development efforts have focused on targeting the cytokine interleukin 1β (IL-1β), which has been shown to be an essential mediator of inflammatory signaling. IL-1β recruits additional cytokines to the site of injury, causing further joint damage. IL-1β signaling is initiated when IL-1β binds to its primary receptor (interleukin 1 receptor 1; IL-1R1), and subsequently to its secondary receptor (interleukin 1 receptor accessory protein; IL-1RAcP) (Furman et al., 2006). In stoichiometric terms, targeting IL-1RAcP (Figure 1) may be many orders of magnitude more effective than conventional IL-1β inhibitors such as IL-1Ra, because the cell surface receptor is found in much smaller concentration than the soluble ligand in the injured joint tissue (Sims, 2010).

In our previous work, we developed a novel structural biology technology called protein painting and discovered a hotspot of interaction between IL-1RAcP and the IL-1β/IL-1R1 binary complex (Luchini et al., 2014). This hotspot interface, which contains Arg286 of IL-1RAcP, was shown to be essential for complex formation, such that blocking this residue with an antibody or a synthetic peptide reduced inflammatory signaling through IL-1β in cell lines. However, there were several remaining questions regarding the suitability of Arg286 interface as a drug target scaffold that needed to be answered. First, it is unclear if Arg286 is a high energy driver of complex formation; if it is not, there may be other residues at the interface that are more suitable to target therapeutically. Secondly, it is unclear if inhibition of the Arg286 residue interface *in-vivo* would prove to be an effective strategy. If Arg286 is a key high-energy mediator of complex formation and its inhibition does result in *in-vivo* effects, then additional medicinal chemistry efforts to develop modified peptides with increased potency would be warranted.

In this work, we performed molecular modeling and molecular dynamics (MD) simulations of the interfaces of the IL-1β–IL-1R1—IL-1RAcP complex to probe the potentiality of Arg286 as an interface scaffold to target with peptide mimetics. We showed that Arg286 of IL-1RAcP is the highest-energy mediator of complex interactions. We further confirmed the role of Arg286 in complex interface stability by blocking Arg286 with a monoclonal antibody that effectively reduced the number of infiltrating immune cells to the site of joint injury in a mouse model of osteoarthritis. Together, computational and animal model work validated Arg286 as a novel and effective drug target for osteoarthritis. Following this validation, we showed that inhibitory peptides mimicking the IL-1RAcP Arg286 region were effective in both protein interaction and cell models and represent scaffolds for further small molecule inhibitor development. The most potent peptide inhibitor showed picomolar activity in a protein pull down assay and is a lead candidate for future inhibitor development. These results offer a more comprehensive understanding of the IL-1β complex, druggable surfaces of the complex, and validate Arg286 as a therapeutic drug target for OA.

## Experimental Methods

### System Construction and MD Simulations of the IL-1β Ternary Complex

Chimera 1.11.2 was used for initial structure editing and visualization (Pettersen et al., 2004). The Amber14 (Pettersen et al., 2004; Case et al., 2014) software suite was used to build and perform all MD simulations and analysis. GAMESS, released April 20, 2017 was used for all QM calculations. The initial IL-1β ternary complex structure was obtained from PDB ID: 4DEP (Thomas et al., 2012). Chimera was used to add hydrogens, fuse sequence gaps, and, using the Chimera Modeler plug-in, to refine loop structures. This refined structure was then used as the starting structure to build a solvated system of the IL-1β ternary complex. tLeaP was used to create the Amber topology and the Amber ff14SB (Maier et al., 2015) force field was used. The system was solvated using the TIP3P water model in a 12.0 Å box, and the overall −2.0 net charge was neutralized with sodium ions. Coordinate and topology files were saved in solvated and unsolvated forms for the following structure files: total complex, IL-1β/IL-1RI, and IL-1RAcP, for use in MM-PBSA/GBSA calculations System minimization was performed using the steepest decent method. Three replicates were created using a random starting velocity at the onset of each equilibration. Equilibration was performed in a two-step, sequential NVT and NPT ensemble. NVT was performed for 10 ps (5,000 steps) at 300 K with NPT performed for 100 ps (50,000 steps) at 1 bar using a Berendsen barostat (Berendsen et al., 1984). Following equilibration, unrestrained production simulations were performed using the Berendsen weak temperature coupling algorithm. Long range electrostatic interactions were calculated using the particle-mesh Ewald (PME) method using cubic interpolation (Essmann et al., 1995). The non-bonded interaction cutoff was set to 1.0 nm and hydrogen bond lengths were constrained with the SHAKE algorithm (Götz et al., 2012). Production simulations were run for 100 ns, for a total of 300 ns of simulation time to evaluate changes in complex interface interactions. Analysis was performed using Amber 14 analysis suite tools, including cpptraj to concatenate and correct the simulation for periodicity. Clustering analysis was performed over the last 20 ns of each replicate and of all replicates combined using the K-means method.

MM-GBSA calculations were performed using per-residue decomposition. The MM-GBSA results were decomposed to electrostatic (ES), van der Waals (VDW), and polar (P) and non-polar (NP) solvation energies as shown in the equation below:

(1) $$\begin{array}{r} \Delta G_{binding}=\Delta G^{ES}+{\Delta G}^{VDW}+{\Delta G}^{P,solv}+{\Delta G}^{NP,solv} \end{array}$$

MM-GBSA calculations were performed for each replicate, over the duration of the simulation and averaged to represent a residue score for the following residues: Gln14, Gln126, Gly140, Gln141, Asp142, Ile143 (IL-1β), Val160, Lys161, Asp162, Arg163, Leu164, Ile165, Asn216 (IL-1R1), and Ile135, Asn168, Leu180, Ile181, Pro245, Arg286 (IL-1RAcP). Those residues were tested because they are residues of interest that contribute the most to binding stability within four possible binding domains (Table 1). All simulations were performed on the George Mason University ARGO supercomputer cluster^1^ (reference https://orc.gmu.edu/research-computing/argo-cluster/argo-hardware-specs/).

**Table 1:** MM-PBSA/GBSA binding regions and specific residues belonging to each.

**Region**	**Residues**	**Protein**
I	Arg-286	IL-1RAcP
	Asp-54, Lys-55	IL-1β
	Thr-437	IL-1RI
II	Asn-219	IL-1RAcP
	Pro-206	IL-1RI
III	Lys-218	IL-1RAcP
	Asp-120	IL-1β
IV	Gln-141, Asp-142, Ile-143, Gln-126	IL-1β
	Asn-166, Phe-167, Asn-168, Gln-165	IL-1RAcP
	Asn-136	IL-1RI

Facio and GAMESS software were used to performed QM calculations. The Fragment Molecular Orbital (FMO) method applies QM calculations to a small subsection of a protein system. Pair interaction energy decomposition analysis (PIEDA) was used to determine interaction energy between pairs of protein fragments. PIEDA was performed by solving for electrostatic (ES), exchange repulsion (EX), and dispersion energy (DI) separately. Then, charge transfer energy (CT) and the mixed term contribution account for the remainder of total energy were calculated according to the following equation:

(2) $$\begin{array}{r} \Delta E^{int}=\Delta E^{es}+\Delta E^{di}+\Delta E^{ct}+\Delta E^{ex} \end{array}$$

### Molecular Modeling and Fingerprint Analysis

Schrodinger Maestro (v. 12.3) (Schrödinger, 2020a) was used for identifying and describing protein interfaces of the IL-1β–IL-1R1—IL-1RAcP complex. Structures, including starting structure and each dominant morphology from MD clustering analysis over the last 20 ns, were pre-processed within the Schrodinger-Maestro protein preparation wizard before fingerprinting. Interacting residues were calculated for chains A, B, and C, corresponding to IL-1β, IL-1R1, and IL-1RAcP, respectively. Hydrogen bonding settings were left as the default with a maximum distance of 2.5 angstroms, minimum donor angle of 120°, and minimum receptor angle of 90°. Interaction cutoff values set as default, with a maximum distance between interacting heavy atoms of 4.0 angstroms and a maximum distance between interactions with hydrogen atoms of 2.5 angstroms. Exported interaction fingerprint data was then processed by an in-house Python (v. 3.8.6) script to be exported in table format (Kozakov et al., 2006). Visualization was performed in PyMOL (v. 2.3.2) (Schrödinger, 2020b). Scripts and input files are housed on our Open Science Framework page (Brown et al., 2017; https://osf.io/82n73/).

### Antibody Design and Rationale

The monoclonal antibodies were custom-produced by Promab, Inc. via immunization of Balb/c mice with Peptide 1.0 (TINESISHSRTEDETRTQILS). An ELISA evaluation of the titer was used to select cells for fusion. Mouse splenocytes with the best titer results were fused with myeloma cells in order to create hybridomas. Western blot against the IL-1RAcP was performed for each selected clone to determine the clones with the highest affinity. Monoclonal antibodies from the selected clones were purified for further *in-vitro* testing. The purification procedure began by priming the mice with 0.5 ml of pristine for 1–2 weeks followed by injection of selected clones. Ascites were then harvested and centrifuged to remove cells and debris then passed over immobilized Protein G columns. Antibodies were washed repeatedly then eluted from the columns in 0.5–1 ml fractions. The fractions were brought to physiological pH, pooled, and dialyzed to ensure a purified product. Two final antibodies were examined, referred to by their clone numbers as 6D3H5 mAb or 7B8D7 mAb.

### Dynabeads Antibody Coupling and Immunoprecipitation

Monoclonal antibodies were coupled to superparamagnetic Dynabeads using Dynabeads Antibody Coupling Kit (ThermoFisher, catalog #14311D). In total, 5 mg of Dynabeads were coupled with 0.75 mg of the 7B8D7 or D3H55 monoclonal antibody as per the instructions in the kit. Once the beads were coupled and re-suspended, the antibody-coupled Dynabeads were stored at 4°C until use. Ten microliter of anti-IL-1RAcP antibody conjugated Dynabeads were incubated with 0, 50, or 100 ng of IL-1RAcP in 50 μL of PBS for 1 h prior to elution by boiling in 20 μL of elution buffer for 5 min. Samples were then run on a SDS-PAGE gel, transferred to a PVDF membrane using western blotting techniques.

### Protein Painting of 7B8D7 Monoclonal Antibody With IL-1RAcP

Recombinant IL-1RAcP and 7B8D7 monoclonal antibody were diluted to respective concentrations of 12.5 μM and using 1x PBS. Equimolar concentrations of proteins were added into a sterile 1.5 mL microcentrifuge tube to create a combination mixture and left to rotate at room temperature for 1 h. Enough of each protein for 1 μg per sample was aliquoted into separate tubes and left to rotate a room temperature for 1 h. During incubation, 1 mg of each dye powder was weighed, placed in an individual sterile tube, and dissolved in 1 mL of 1x PBS. Mini quick-spin oligo columns (Sigma, Cat. No. 11814397001) were prepared by mixing the matrix thoroughly via inversion then centrifuged at 1,000 × g for 1 min. Approximately 300 μl of dH_2_O was then placed into the center of the column bed to wash size exclusion media. A total of 1 μl of the protein complex or individual proteins was added to 50 μl of 1x PBS as a control or 50 μl of each type of dye, and incubated for 5 min. During this incubation, the columns were centrifuged at 1,000 × g for 2 min to remove the dH_2_0 wash. Following centrifugation, the entire contents of the protein and dye samples (51 μl) were placed into the center of the corresponding column and centrifuged at 1,000 × g for 1 min to remove unbound paint molecules from the painted samples.

### Mass Spectrometry Sample Preparation

Protein painting samples were denatured with 2 M urea and 100 mM DTT for 15 min in a 37°C, then alkylated with 25 mM iodoacetamide for 15 min in the dark. Samples were digested with the addition of 50 mM (NH_4_)_2_CO_3_ to maintain pH and a final concentration of 1:10 wt/wt of trypsin to sample at 37°C for 1.5 h. Digestion was halted with 2% w/v acetic acid.

Desalting of the samples was performed using Zip Tip® Pipette Tips or Pierce® C18 Spin Columns. During the Zip Tip procedure, each pipette tip was activated via washing with Buffer B (0.1% TFA, 80% acetonitrile) two times, followed by washed Buffer A (0.1% TFA) three times. Each sample was passed through the Zip Tip and dispensed in an empty sterile microcentrifuge tube twice. The sample bound to the Zip Tip resin was then washed with Buffer A four times, and eluted twice with Buffer B. Desalting via the C18 Spin Column was completed as per the instructions included with the Pierce C18 Spin Columns Eluted samples. Samples were dried under nitrogen evaporation and analyzed by reversed-phase liquid chromatography nanospray tandem mass spectrometry (LC-MS/MS) using an LTQ-Orbitrap mass spectrometer (ThermoFisher).

### Mass Spectrometry Analysis

After sample injection by autosampler, the C18 column (0.2 × 50 mm, Michrom Bioresources, Inc.) was washed for 2 min with mobile phase A (0.1% formic acid) and peptides were eluted using a linear gradient of 0% mobile phase B (0.1% formic acid, 80% acetonitrile) to 50% mobile phase B in 40 min at 500 nanoliter/min, then to 100% mobile phase B for an additional 5 min. The LTQ Orbitrap mass spectrometer was operated in a data-dependent mode in which each full MS scan was followed by five MS/MS scans where the five most abundant molecular ions were dynamically selected for collision-induced dissociation (CID) using a normalized collision energy of 35%. Tandem mass spectra were searched against the NCBI human database with SEQUEST using tryptic cleavage constraints. High-confidence peptide identifications were obtained by applying the following filter criteria to the search results: Xcorr vs. charge ≥1.9, 2.2, 3.5 for 1+, 2+, 3+ ions; ΔCn >0.1; probability of randomized identification ≤0.01.

### Development of Animal Model of IL-1β-Induced Inflammation

All protocols involving mice were approved by the Institutional Animal Care and Use Committee (IACUC) at George Mason University, Fairfax, VA. Female DBA/2J mice (*n* = 15) from Jackson Laboratories were procured at 8 weeks of age and housed until 16 weeks of age, when peak bone mass is achieved and active growth has decreased (Götz et al., 2012; see text footnote 1). The animals were then divided into groups and received subcutaneous injections in the foot pad. One group (*n* = 3) of mice received a simultaneous subcutaneous injection of IL-1β dissolved in PBS plus 0.01% endotoxin-free BSA, as a positive control, while a second group of mice (*n* = 3) received a subcutaneous injection of PBS plus 0.01% endotoxin-free BSA as a negative control. The three remaining treatment groups (*n* = 3 per group) received injections containing either 10, 50, or 250 ng of IL-1β dissolved in PBS plus 0.01% endotoxin-free BSA. All animals were sacrificed at 4 h post-injection (when IL-1β has been shown to be at peak activity) (Schrödinger, 2020a). Tissue from the hind footpads was fixed in 10% buffered formalin, paraffin embedded, cut into 5 μM section, and stained with hematoxylin, eosin (Emmert-Buck et al., 1996) and either anti-PMN Ab (a-Ly6G) to observe the number of migrated granulocytes or 7B8D7 anti-IL-1RAcP mAb to validate role of IL-1RAcP in mouse model.

### Antibody Inhibition in the Animal Model of IL-1β Induced Inflammation

Female DBA/2J mice (*n* = 25) from Jackson Laboratories were procured at 8 weeks of age and housed until 16 weeks of age. The animals were then divided into five groups and received simultaneous subcutaneous injections into the ankle joint. The positive control group of mice (*n* = 5) received an injection containing 50 ng of IL-1β dissolved in PBS plus 0.01% endotoxin-free BSA. The mice within the negative control group (*n* = 5) received a subcutaneous injection of PBS plus 0.01% endotoxin-free BSA. The three remaining groups (*n* = 5 per group) received injections containing either 3 nM of 6D3H5 mAb, 3 nM of 7B8D7 mAb, or 1.34 nM of 7B8D7 mAb dissolved in PBS plus 0.01% endotoxin-free BSA and 50 ng of IL-1β. All animals were sacrificed at 4 h post-injection. Tissue from the ankle joint was fixed in 10% buffered formalin, paraffin embedded, and staining with hematoxylin, eosin, and anti-PMN Ab (a-Ly6G) to observe the number of migrated granulocytes. *P*-values were calculated using 1-way ANOVA with Dunnett's Multiple Comparison's test.

### Peptide Production

Peptides 1.0, 1.1, 1.2, 2.0, 3.0, 4.0, and 4.3 were custom produced by Peptide 2.0, Inc. Peptide 4.1 and 4.2 were custom produced by Biomatik. Both companies utilized Fmoc/tBu solid-phase synthesis procedures to prepare peptides. Peptides were purified via RP-HPLC to 98% purity, then lyophilized. Sequence was confirmed by MS. Length and sequence of the lead peptide (Peptide 1.0) was chosen according to the following principles: (1) natural sequence of IL-1RAcP in close juxtaposition with IL-1β and IL-1RI as identified with protein painting, (2) sequence included the Arg286 residue in its central region, (3) sequence was compatible with standard solid-phase peptide synthesis methods, and (4) hydrophilicity properties (33% charged and 19% hydrophobic residues) suggested solubility in aqueous solutions. Cysteine was introduced in substitution for isoleucine to afford cyclization via disulfide bridge creation, to restrict conformational flexibility of the molecule. Cysteine was introduced in different positions (282 or 278, and 295) in order to test whether cycle length and position would influence biological activity (Peptide 1.1, 1.2, 4.1, 4.2, and 4.3). In the negative control Peptide 2.0, alanine was substituted to arginine in position 286 to determine arginine importance in biological activity. Asparagine and glutamine were substituted for aspartic acid and glutamic acid in order to test the influence of charge distribution on peptide binding properties (Peptides 4.1, 4.2, and 4.3). Methionine was substituted for Threonine in position 293 to strengthen peptide secondary interaction with the of IL-1RI scaffold of aromatic residues and enhance peptide multivalency.

### Receptor Complex Immunoprecipitation Pull-Down

IL-1β (20 ng μl^−1^), IL-1RI (100 ng μl^−1^), and 6xHis-tagged IL-1RAcP (40 ng μl^−1^) were incubated with parent Peptide 1.0, a peptide variant, or the antibody inhibitor at various concentrations in 50 μl of PBS for 1 h at room temperature under rotation. In parallel, IL-1β, IL-1RI, and 6xHis-tagged IL-1RAcP were allowed to interact without parent Peptide 1.0 as a positive control. IL-1β by itself acted as a negative control. BcMag Protein G Magnetic Beads (50 μl, Bioclone) were washed 3 times with washing buffer (57.7 mM Na_2_HPO_4_, 42.3 mM NaH_2_PO_4_, pH 7.0), incubated with anti-6xHis mouse monoclonal antibody (1 μg, Abcam) for 30 min under rotation, isolated with neodymium magnets, and washed three times with washing buffer (57.7 mM Na_2_HPO_4_, 42.3 mM NaH_2_PO_4_, pH 7.0). Protein complex mixtures were incubated with the anti-His antibody decorated magnetic beads for 1 h at room temperature under rotation. Magnetic beads were separated (neodymium magnets) and washed 3X with buffer (57.7 mM Na_2_HPO_4_, 42.3 mM NaH_2_PO_4_, pH 7.0). Immunoprecipitated proteins were eluted with 20 μl of 4X sample buffer (10 min, 70°C) and analyzed by western blotting (anti-IL1β antibody).

### Western Blotting

SDS-PAGE was used to separate the immune-precipitated proteins in a 4–20% Tris-Glycine gel for 90 min at 125 V. The proteins were transferred for 2 h at 25 V onto an Immobilion PVDF membrane. After blocking the membrane using PBS containing 0.2% (*w/v*) I-Block and 0.1% Tween 20 for 1 h at room temperature (or overnight at 4°C), the membrane was subjected to a 2-h incubation with an anti-IL1β antibody (1:1,000, Cell Signaling Technology) while undergoing continuous agitation. The membrane was washed with PBS containing 0.1% Tween 20, then subjected to a 1-h incubation with the secondary anti-mouse horseradish-peroxidase-conjugated anti-IgG antibody (1:10,000, Invitrogen). The western blot was developed using a chemiluminescence system (SuperSignal^TM^ West Dura Extended Duration Substrate) and imaged using a Kodak 4000MM system.

### Peptide Inhibitor Testing in Cell Cultures

HEK 293 reporter cells for human IL-1β (HEK-Blue IL-1β cells, Invivogen) were used to test the ability of peptide variants to inhibit IL-1β signaling pathway. HEK-Blue IL-1β cells were used according to manufacturer instructions. HEK-Blue IL-1β cells were cultured in Dulbecco's modified Eagle's medium (DMEM, Gibco) additioned with 10% heat inactivated fetal bovine serum (ATCC), 4.5 g/l glucose (Sigma), and 2 mM L-glutamine (ATCC) at 37°C, 5% CO_2_, in a humidified environment. HEK-Blue IL-1β cells (5,000 cells/well) were plated into 96-well tissue culture plates. The following day, cells were pre-treated for 1 h with peptide variants (Peptides 1.0, 1.1, 1.2, 2.0, 3.0, 4.0, and 4.3) or soluble IL-1RAcP (2 ng/μL) as a positive control. The cells were then stimulated with IL-1β at 2.5 ng/ml for 30 min. Following stimulation, the medium was changed and the inhibitors were added again to the plate. The cells were then left to incubate overnight. The next day, 160 μl of QUANTI-Blue (Invivogen) were added to each well. The plate was incubated at 37°C for 1 h. Then, the plate was placed in a spectrophotometer to detect the levels of secreted embryonic alkaline phosphatase (SEAP) in each well at a wavelength of 625–655 nm. Each peptide was tested in triplicate and an ANOVA test with Dunnett's test for multiple comparisons was performed to determine significance of inhibition.

#### Statistical Analysis

Statistical tests (One-way ANOVA with Dunnett's Multiple Comparison adjustment) and non-linear regression of the dose response pull down peptide inhibition data were performed using GraphPad Prism.

## Results

### MD Simulations of Ternary Complex and Interaction Interface Analysis

In order to identify hot spots for interface targeting via modeling and to determine interactions critical to binding and activity, a computational study of the ternary IL-1β complex interaction was performed through classical MD simulations and quantum mechanical calculations. MD simulations were performed to determine a dominant morphology of the ternary complex after allowing the structure to readjust for solvent present and to determine and assess subunit interfaces after simulation. Root-mean-square deviation (RMSD), radius of gyration, root-mean-square fluctuation (RMSF), and potential energy, were calculated in each replicate simulation (Supplementary Figures 1–3). Backbone atom RMSD was performed to determine the average distance from the original structure to the current frame, and for the last 50 ns of each simulation, demonstrated <0.1 nm of deviation of current frames relevant to the starting structure. RMSD clustering was also performed over the last 50 and 20 ns of the simulation to obtain a dominant morphology to use in subsequent fingerprinting analysis (Supplementary Figure 4). Clustering analysis indicated a conserved dominant cluster that represented more than 30% of frames during the last 20 ns and only differed from the second most populated cluster by less 0.3 nm. Coupled with fingerprint analysis (discussed below), we wanted to determine if any new interactions were developed over the course of the simulation as well as analyze consistency in interactions from the crystal structure. RMSD (Supplementary Figure 1), RMSD clustering (Supplementary Figure 4), and fingerprinting (Supplementary Tables 1–13) established that there were minimal fluctuations in interface interactions and no major changes in secondary structure, providing a representative structure to use for further analysis and as an initial probe at studying the dynamic nature of the IL-1β–IL-1R1—IL-1RAcP complex.

To further determine if any subunit of the ternary complex moved away from or in a major location different than the starting structure, radius of gyration (Rg), and root-mean-square fluctuation (RMSF) analysis was performed. Rg refers to the root-mean-square distance of atoms from their mass-weighted center and highlighted that the structure remained compact, between 3.1–3.2 nm as complex throughout the duration of the simulation (Supplementary Figure 2). This highlights that there were likely minimal major structural changes throughout the simulation, and was in agreement with RMSF and RMSF clustering data that demonstrated minimal peeks in fluctuation, with the most fluctuation (<0.4 nm) at residue ranges 175–200, 350–375, 450–460, 500–510, and 680–705 (residues labeled in order of subunits of the ternary complex—IL-1β–IL-1R1—IL-1RAcP) (Supplementary Figure 3). These residue ranges are primarily in loop structures and are not at the interaction interface. Additional fluctuation was observed at the termini, notably the C-terminal, which is expected due to standard protein termini flexibility. Collectively, over the simulation, there was not major structural rearrangement. There was moderate movement in some regions to adjust for presence of solvent and to achieve a more energetically favorable positioning, but overall, for the purposes of this work, we have dominant morphologies of the IL-1β–IL-1R1—IL-1RAcP complex to probe with more in-depth analysis of interface interaction changes as a result of dynamics and to hypothesize the mechanism of inhibition by designed inhibitors.

Fingerprint analysis was performed on the starting structure and dominant morphology of each replicate to determine changes in residues participating in subunit interface interactions (Supplementary Tables 1–13). The majority of interactions, ~80%+, that were observed in the crystal/starting structure were retained during the duration of the simulation and are observed in the dominant morphology of the last 20 ns of each replicate. Specifically, hydrogen bonds between IL-1RAcP:ASN168—IL-1β:GLY140 and IL-1RAcP:SER185—IL-1β:ASP145, a salt bridge between IL-1RAcP:ARG286—IL-1β:ASP54, and an aromatic interaction between IL-1RAcP:PHE167—IL-1β:GLN141 were all retained between each replicate (Supplementary Table 1 and the starting structure). The salt bridge between the previously identified key residue IL-1RAcP:ARG286 and IL-1β:ASP54 also appeared to have moderate affinity and interaction with another nearby negatively charged amino acid IL-1β:GLU111, suggesting it may play a role in providing a negative binding site for IL-1RAcP:ARG286 (Supplementary Figure 4), further highlighting the influence of Arg286. Full interaction fingerprint data can be found within the Supplementary Tables 2–13. Simulation results indicate a highly maintained interaction network between the IL-1β–IL-1R1—IL-1RAcP subunits, suggesting that both dual subunit and individual subunit inhibitors can be designed and insight into the consistent and transient interactions.

### MM- GBSA and Fragment Molecular Orbital (FMO) Results Confirm That IL-1RAcP Arg286 Is a Hot Spot of Interaction

**Figure 2:**
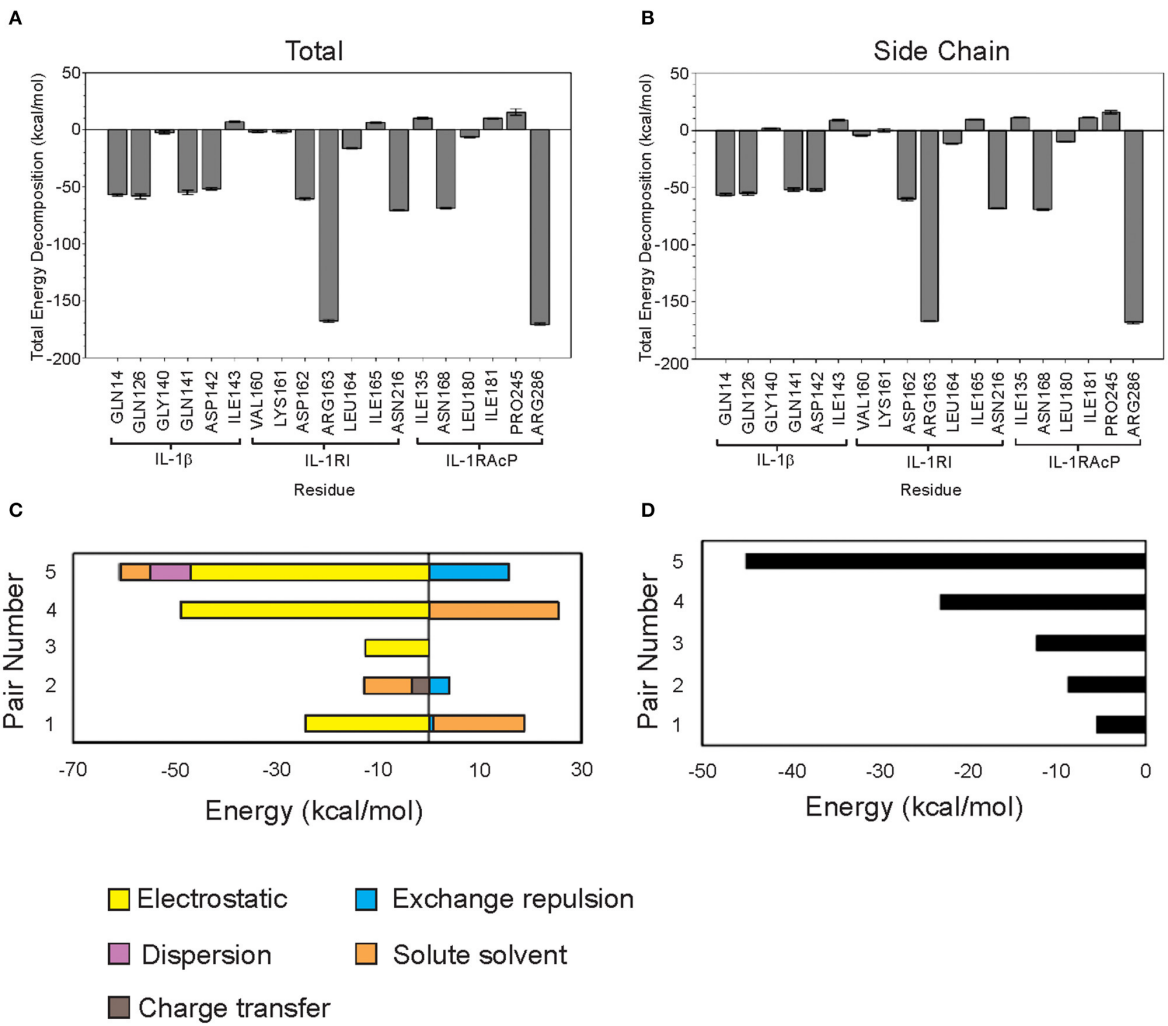
Classical and quantum mechanics calculations show that Arg286 of IL-1RAcP is the highest energy mediator of IL-1β:IL-1RI:IL-1RAcP complex formation. **(A)** MM-PBSA/GBSA decomposed free energy of binding for region I-IV residues. **(B)** MM-PBSA/GBSA total free energy of binding for region I-IV residues. **(C)** FMO Decomposed pair interaction energy for pairs 1–5. **(D)** FMO Total pair interaction energy for pairs 1–5.

Four possible binding regions were chosen through literature review (Punzi et al., 2016) and visual inspection of the protein structure (Table 1). MM-GBSA calculations were performed on the labeled residues of interest to determine which binding region, and ultimately which residue potentially contributes the most to binding stability at the interfaces of the IL-1β–IL-1R1—IL-1RAcP complex (Figures 2A,B).

As expected, calculations according to Equation (1) showed that charged residues had large electrostatic contributions. High polar solvation energy was expected in residues with large negative electrostatic contributions to compensate for solvent interactions (Supplementary Table 14).

Arg286 has the lowest total free energy of binding which is dominated by its internal energy contribution (Supplementary Figure 2A). Asp54 is a predicted binding partner due to the residue orientation in the crystal structure (PDB ID: 4DEP) and low ES contribution. Additionally, MD simulation studies showed that there is a negatively charged pocket around both Asp54 and Glu111—further highlighting the influence of Arg286 at mediating this interaction interface (Supplementary Figure 4). Region I is an interesting target for further study on probing and targeting the interfaces of these subunits. Region IV has several residues with low total free energy of binding values but without a dominant type of interaction. This indicates that the interaction among residues is more complex than simply a single electrostatic interaction between Arg286 and Asp54 holding the proteins together (Table 1), which we further confirmed with fingerprinting analysis, which mostly maintained Arg286-Asp54 interaction; but other interactions and a favorable hydrophobic interface also drove position favorability.

Though several residues showed compelling free energy of binding values, Arg286 had the lowest value and dominant internal energy contribution, consistent with previous results identifying this residue as a “hot spot” for protein-protein interaction. Arg286 was ultimately chosen for further study using FMO methods to account for quantum mechanical contributions to binding at the interface.

Residues within 0.5 nm of Arg286 were chosen for FMO calculations. Each terminal residue was manually capped with a methyl group, creating N-met or acetyl for the N- and C-termini, respectively. The Facio software was then used to fragment segments longer than 2 amino acids into 1–2 residue components for the FMO QM calculations. The five lowest energy pair interactions are shown in Figure 2C. Among the five pairs, Arg286 is involved in three separate interactions (Table 2). Electrostatic and charge transfer interactions were dominant in those three pairs, as expected with a negatively charged residue. Pair 5 had lowest total interaction energy, composed mainly of electrostatic forces. This is likely due to an interaction between Arg286 of fragment 1 and Asp54 of fragment 2 (Figure 2D). Hydrogen bond analysis in Chimera and in finger print analysis identified a hydrogen bond between Arg286 and Asp54. Hydrogen bonds are predominantly electrostatic in nature, and they would contribute to the large electrostatic component of the Arg286:Asp54 pair interaction energy seen in MM-PBSA/GBSA and FMO calculations. The interaction between Arg286 and Asp54 was suggested to be predominantly a salt bridge (Günther et al., 2017), so we expect the hydrogen bond to give a lesser contribution. Combining the MMGBSA and FMO analysis, we have indicators at energy hot spot interactions that influence the complex interaction network. Disrupting these specific amino acid interactions with an inhibitor that specifically targets areas, with mimicking physicochemical properties, is a next step for disruption of complex formation.

**Table 2:** Residues belonging to pairs 1-5 in FMO calculations.

**Pair number**	**Fragment 1 (IL-1RAcP)**	**Fragment 2 (IL-1β)**
5	Arg-286, Thr-287	Asp-54, Lys-55
4	Arg-286, Thr-287	Glu-111
3	Asn-186, Asn-187	Glu-111
2	Ile-184, Ser-185	Lys-138
1	Arg-286, Thr-287	Glu-105, Ile-106

### IL-1RAcP Targeted Monoclonal Antibody Design and Validation

Computational studies highlighted the influence of Arg286 as a high energy mediator of the ternary IL-1β complex formation, as well as identified persistent and transient residue interactions at the interface of the ternary complex. To further probe the role of Arg286 as a crux point of influence in interface interactions, a monoclonal antibody against the IL-1RAcP Arg286 region was created, and its capability of forming a stable complex with IL-1RAcP was tested via immunoprecipitation and protein painting.

**Figure 3:**
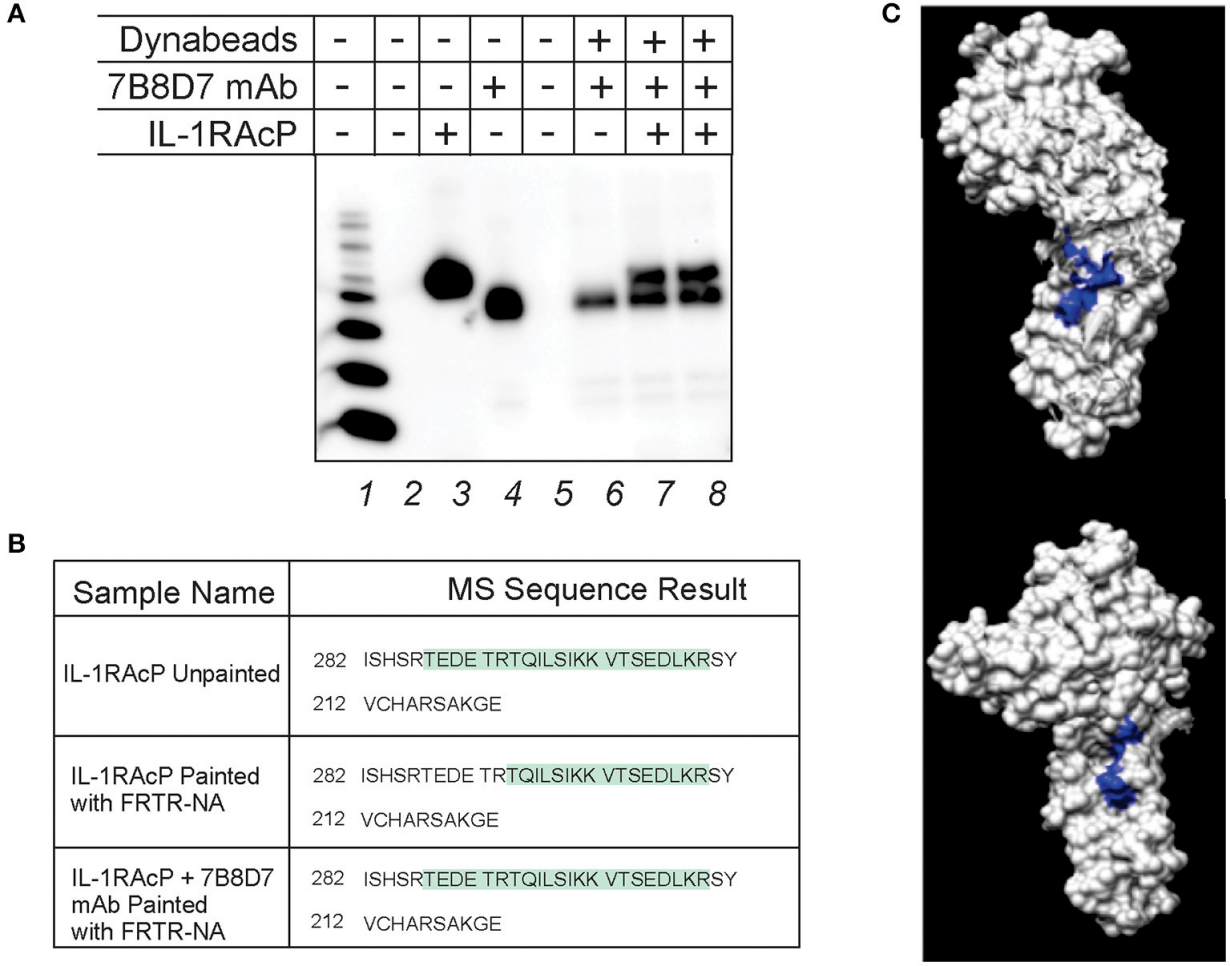
7B8D7 monoclonal antibody forms a stable complex with IL-1RAcP and targets Arg286. **(A)** 7B8D7 monoclonal antibody (mAb) coupled Dynabeads were incubated with IL-1RAcP at various concentrations and eluted by boiling in sample buffer. Lane 1 is the molecular ladder, lane 2 and 5 are empty, lane 3 contains IL-1RAcP without beads, and lane 4 contains 7B8D7 mAb without beads. Lane 6 contains the elution from the antibody-coupled beads without IL-1RAcP, lane 7 contains elution from the antibody-coupled beads with 50 ng of IL-1RAcP, and lane 8 contains elution from the antibody-coupled beads with 100 ng of IL-1RAcP. **(B)** MS data from both the IL-1AcP individually and in complex with 7B8D7 mAb painted with FRTR-NA was compared. The regions in green correspond to amino acid sequence fragments identified by mass spectrometry. The differences between the highlighted regions correspond to the fragments that were solvent excluded, and therefore involved in the protein-protein binding interface. **(C)** The crystal structure of the IL-1RAcP is shown at two different angles (90° counterclockwise from one another) with the missing “TEDETR” fragment highlighted in blue.

The immunoprecipitation was conducted using anti-IL-1RAcP antibody (clone 7B8D7) conjugated Dynabeads that were allowed to incubate with IL-1RAcP. After magnetic separation, samples were analyzed using western blotting. The membrane reported in Figure 3A shows the IL-1RAcP band at the expected 66 kDa molecular weight (Lane 3) and the antibody heavy chain band at approximately 50 kDa (Lane 4). Elution of 7B8D7 conjugated Dynabeads resulted in a 50 kDa band, confirming successful coupling of the antibody to the beads. When the conjugated beads were allowed to interact with IL-1RAcP, the antibody formed a stable complex with IL-1RAcP that resulted in the visualization of both proteins in the pulled-down fraction (Figure 3A, Lanes 7–8). Similar experiments were conducted for clone 6D3H5 and confirmed stable mAb/IL-1RAcP complex formation (Supplementary Figure 5).

Protein painting was performed in order to experimentally confirm mAb epitope binding. IL-1RAcP was incubated with 7B8D7 mAb and then the complex was painted with the FRTR-NA fast dye. In parallel, IL-1RAcP was evaluated individually with and without paints by MS analysis in order to assess the total sequence coverage and where the native internally folded residues are found, respectfully. All samples were then evaluated by MS analysis to look for differences in the Arg286 sequence region where the mAb would bind. The individual unpainted IL-1RAcP sequence coverage demonstrated that the entire peptide fragment including the Arg286 residue was present (Figure 3B, first row). When the uncomplexed IL-1RAcP was painted with the FRTR-NA fast dye, the peptide fragment “TEDETR” was missing from the resulting sequence; this confirms that the peptide is in a solvent accessible area and that the dye binding blocked trypsin digestion rendering the sequence invisible to mass spectrometry. Upon complex formation, post-painting with FRTR-NA, the complete peptide sequence was once again present. This data showed that in presence of the 7B8D7 mAb, the FRTR-NA dye molecules were unable to bind to the “TEDETR” region of the IL-1RAcP, and therefore that this region is in the interface between the protein and the mAb. The TEDETR sequence is a tryptic peptide that extends from IL-1RAcP Thr287 to Arg292 and emerged as an IL-1RAcP interface region in the IL-1—IL-1RI—IL-1RAcP ternary complex via protein painting (Luchini et al., 2014). Clone 6D3H5 binding to the Arg286 epitope was confirmed via peptide competition (Supplementary Figure 5).

### Animal Model Development for Evaluation of 7B8D7 mAb Affinity and Specificity

**Figure 4:**
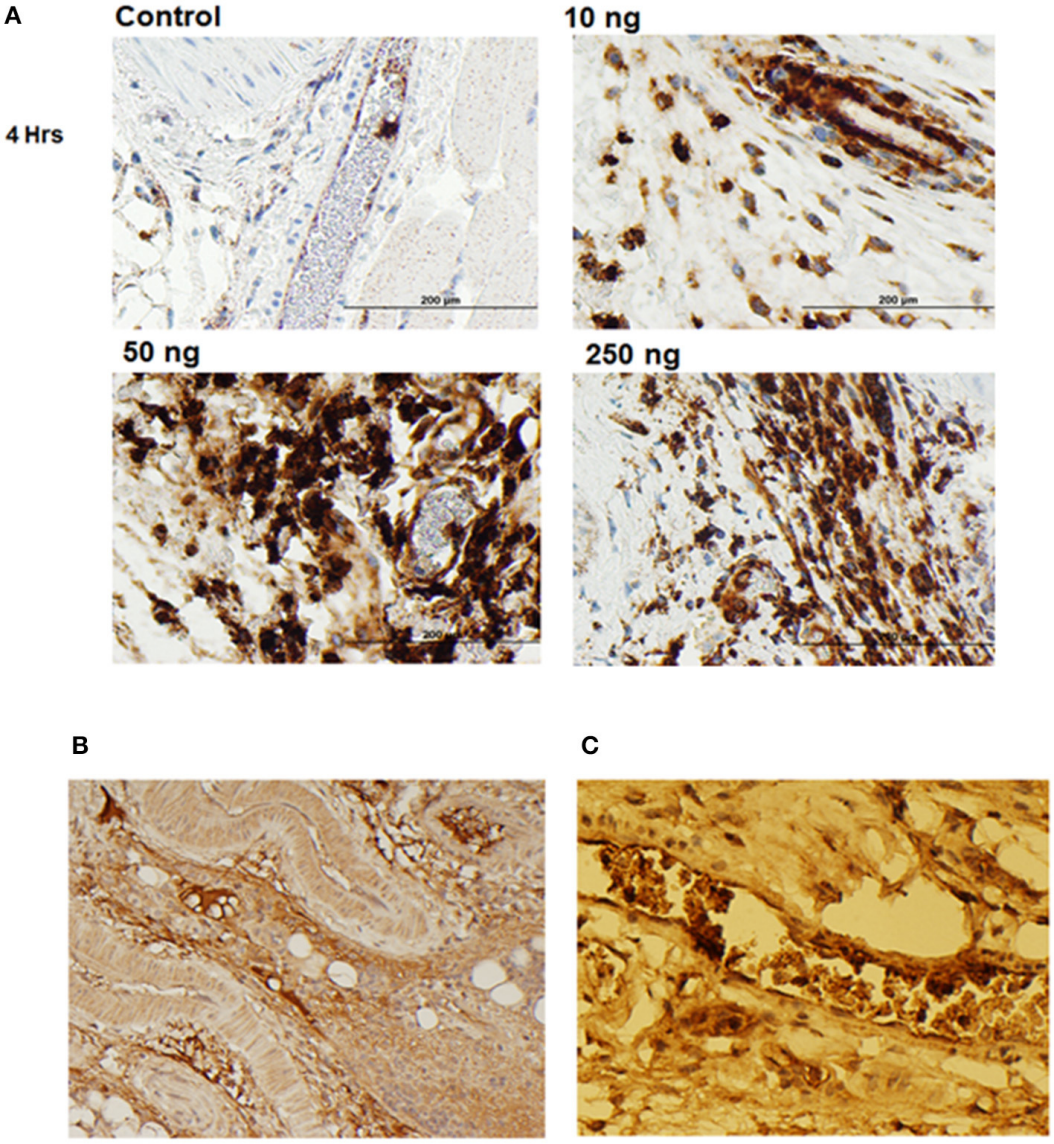
IL-1β foot pad injection induces local granulocyte recruitment as demonstrated by α-Ly6g and α-IL-1RAcP tissue immunostaining. **(A)** Hematoxylin and eosin staining were used, as well as anti-PMN antibody (α-Ly6g) to visualize the granulocytes. The control footpad received an injection of PBS. Injections of 10, 50, or 250 ng of IL-1β dissolved in PBS were administered to three separate footpads. **(B)** Hematoxylin and eosin staining were used, as well as anti-IL-1RAcP antibody (7B8D7) to visualize IL-1RAcP present on the cell surface of the recruited immune cells. The footpad received 50 ng of IL-1β dissolved in PBS plus 0.01% endotoxin-free BSA prior to fixation. Cell surface IL-1RAcP is stained in brown and the cell nuclei are stained in blue. The image was taken at a 10X magnification. **(C)** A 40X magnification view of a blood vessel within the footpad tissue. The immune cells contained within the vessel are stained dark brown, which indicates a high incidence of IL-1RAcP in the region of inflammation.

To further display the importance of the Arg286 hot spot residue to IL-1β complex formation and in the induction of an inflammatory response, an animal model of inflammation was developed for the evaluation of the 7B8D7 mAb *in vivo*. In this model, IL-1β was injected in the footpad of DBA/2J mice and the presence of Ly6g+/IL-1RAcP+ granulocytes was investigated in the footpad tissue as a sign of local inflammation. In a dose optimization study female DBA/2J mice received a subcutaneous injection of 10, 50, or 250 ng of IL-1β dissolved in 50 μl PBS into the hind footpads. The control mice received a subcutaneous injection of PBS alone. All animals were sacrificed at 4 and 24 h post-injection. In the footpad tissue from the control group, a limited number of granulocytes were observed (Figure 4A). IL-1β injection induced localized inflammation seen via granulocyte recruitment, and the optimum IL-1β dose for recruitment was 50 ng (Figure 4A). The footpad tissue was also stained with the 7B8D7 mAb and the IL-1RAcP became clearly visible on the surfaces of the recruited granulocytes (Figure 4B). Figure 4C shows a tissue section containing a blood vessel with granulocytes moving toward the area of induced inflammation. The deep brown staining on the cells located within the blood vessel indicated a high incidence of IL-1RAcP, as well as high specificity of binding of the 7B8D7 mAb (Figure 4C).

The ability to induce localized inflammation makes this an ideal animal model to test the functionality of the 7B8D7 mAb.

### Monoclonal Antibodies Demonstrates Potent Inhibition *in vivo*

**Figure 5:**
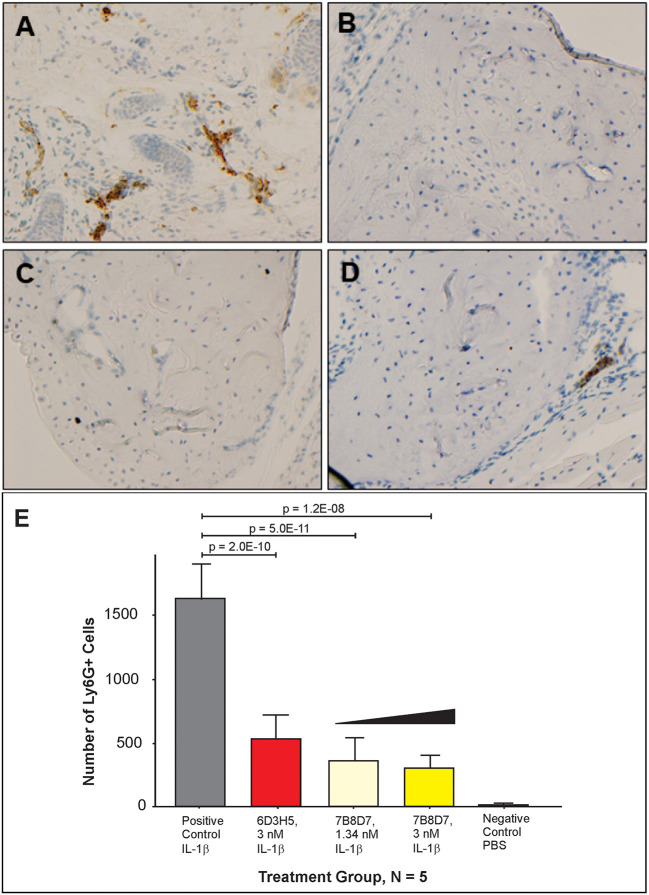
Arg286 targeted mAbs display significant inhibition of inflammation *in vivo*. Injections of either 3 nM of 6D3H5 mAb, 3 nM of 7B8D7 mAb, or 1.34 nM of 7B8D7 mAb dissolved in 50 μL of PBS (containing 50 ng of IL-1β) were administered into the ankle joint of three separate groups of mice. The positive control group received an injection of 50 ng of IL-1β, while the negative control footpad received an injection of only PBS. Hematoxylin and eosin staining were used, as well as anti-PMN antibody (α-Ly6g) to visualize the granulocytes for **(A)** the positive control, **(B)** the negative control, **(C)** 3 nM and 1.34 nM 7B8D7 mAb, and **(D)** 3 nM 6D3H5 mAb. **(E)** The number Ly6G+ granulocytes were calculated as a marker of inflammation. *P*-values were calculated using 1-way ANOVA with Dunnett's Multiple Comparison's test.

The mouse animal model was used to demonstrate that anti-Arg286 antibodies are effective in attenuating IL-1β induced inflammation *in vivo*. Two anti-Arg286 mAb clones were tested: 6D3H5 and 7B8D7. Female DBA/2J mice received an injection of 3 nM of 6D3H5 mAb, 3 nM of 7B8D7 mAb, or 1.34 nM of 7B8D7 mAb dissolved in PBS containing 50 ng of IL-1β into the ankle joint. The mice within the positive and negative control groups received an injection of IL-1β in PBS and PBS alone, respectively. All tissues were stained in the same manner as the prior mouse model experiment and the number of Ly6G^+^ cells were counted as a marker of inflammation (Figures 5A–D). There was a significant difference in granulocyte recruitment between the treatment groups and the positive control. At 3 and 1.34 nM of the 7B8D7 mAb, the number of invading Ly6G^+^ cells present in the tissue was 81% (*p* = 1.2E-8) and 78% (*p* = 5E-11) less than that of the positive control (Figure 5E). A 67% (*p* = 2E-10) decrease in granulocyte recruitment was also detected when 3 nM of the 6D3H5 mAb was administered. No significant difference was seen between the treatment groups themselves. This confirms that the Arg286 hot spot is an effective potential drug target against OA in an *in vivo* model.

### Modified Derivatives of Peptide 1.0 Validate the Importance of Arg286 Residue

On the basis of protein painting data (Luchini et al., 2014), a synthetic peptide mimicking IL-1RAcP Arg286 hot spot region (Peptide 1.0) was created. The structure of the lead inhibitor sequence TINESISHSRTEDETRTQILS is derived from a beta-hairpin secondary structure in an anti-parallel beta sheet of the IL-1RAcP protein (Table 3). The crystal structure of the IL-1 receptor-signaling complex and molecular modeling suggest that the Arg286 residue of IL-1RAcP interacts with the Asp54 residue of IL-1β. This interaction makes Peptide 1.0 a very strong candidate to inhibit IL-1β complex formation and validate the overall importance of the Arg286 hot spot to complex formation *in vitro*. Peptide 1.0 variants were obtained by amino acid substitutions and cyclized motifs in order to increase peptide structural stability and potency (Table 3).

**Table 3:** Peptide 1.0 and its variant sequences.

**Peptide name**	**Peptide sequence**	**Peptide length**	**Cycle length**
1.0	TINESISHSRTEDETRTQILS	21	0
1.1	TINESCSHSRTEDETRTQCLS	21	14
1.2	TCNESISHSRTEDETRTQCLS	21	18
2.0	TINESISHSATEDETRTQILS	21	0
3.0	TINESISHSRTEDETRMQILS	21	0
4.0	TINQSISHSRTQNQTRTQILS	21	0
4.1	TINQSCSHSRTQNQTRTQCLS	21	14
4.2	TCNQSISHSRTQNQTRTQCLS	21	18
4.3	TINQCSHSRTQNQTRTQCLS	20	14

*Amino acid substitutions and introduction of cyclized motifs are highlighted in red. The Arg286 position is shown in green. The mass spectrometry datasets presented in this study can be found in online repositories. The name of the repository and accession number can be found at: MassIVE MSV000086480*.

Substitution of glutamine for glutamic acid causes a change in charge distribution, which may assist in tighter binding by the peptides. Cyclization of peptides has proven to be a successful strategy for increasing peptide stability against proteolytic degradation. In addition, cyclization affords reduced conformational freedom and can thereby reduce entropic costs for binding to the target (Goodwin et al., 2012). Since the X-ray crystal structure of IL-1RAcP showed that the Peptide 1.0 is tightly packed when bound in the 3-way complex, this positions the end groups in close enough proximity to promote cyclization. Substitution of two cysteines for isoleucines in the original Peptide 1.0 sequence encourages cyclization. Ring size optimization can be affected by the position of the two allylated cysteine residues in the peptide sequence. Substitution of methionine for threonine (Peptide 3.0) aimed to strengthen peptide secondary interaction with the Arg208 region of IL-1RI.

### Characterization Was Performed Using *in vitro* Pull-Down Assays and Interleukin Signaling in Cell Lines

**Figure 6:**
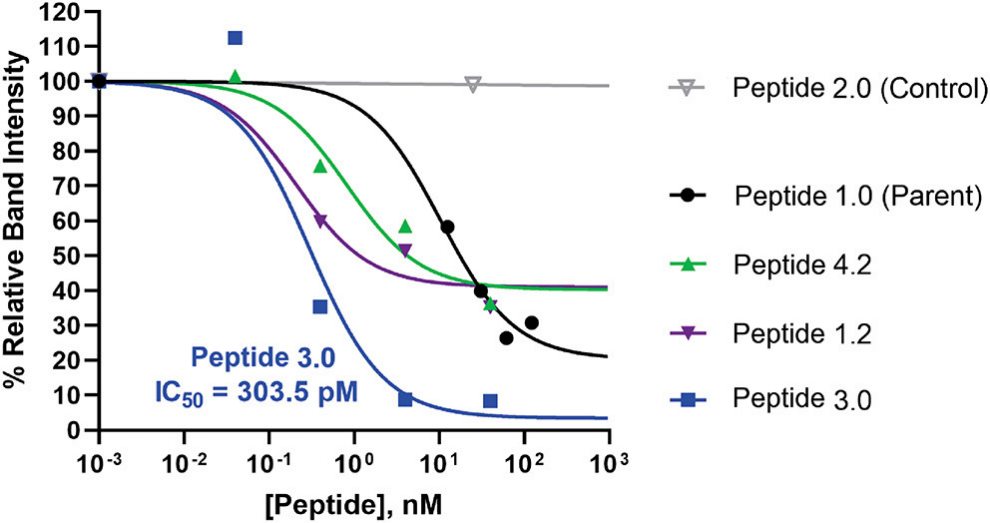
Comparison of peptide variant inhibition of IL-1β complex formation using IL-1RAcP pull-down assay and IL-1β western blot staining. Dose response pull down assays were performed, whereby Dynabeads were coupled to IL-1RAcP and were allowed to interact with IL-1β and IL-1RI in presence and absence of peptide inhibitors. Peptide sequences are reported in Table 3. Peptide 2.0 is a negative control in which Arg286 was substituted with Alanine. Western blot band intensities were calculated using ImageJ analysis software for each of the peptide inhibitors. These intensity values were then divided by the band intensity value of the positive control to determine the relative band intensity. This value correlates with percent inhibition of IL-1β complex formation. The *p*-values were calculated with a 1-way Anova using Dunnett's test for multiple comparisons. The IC50 was calculated for the most potent peptide inhibitor (peptide 3.0).

In order to ascertain the peptide effectiveness in disrupting IL-1β complex formation *in vitro*, receptor complex immunoprecipitation pull-down and western blot assays were performed. Relative band intensities of the immunoprecipitation pull-down samples were calculated as a ratio of (precipitated IL-1β in presence of peptide inhibitor)/(precipitated IL-1β in absence of peptide inhibitor) (Figure 6 and Supplementary Figure 6). As seen in Figure 6, all concentrations of Peptide 1.0 displayed some degree of inhibition on the IL-1β complex formation. Higher Peptide 1.0 concentrations (such as 124 and 62 nM) resulted in a 26–30% reduction of IL-1β pull down, while lower concentrations (such as 12.5 nM) resulted in 60% IL-1β pull down (Figure 6).

Peptide 3.0 which was obtained by substituting a methionine to threonine in position 293, achieved the best performance among all tested peptide variants. Higher concentrations of the Peptide 3.0 (40 and 4 nM) reduced IL-1β pull down to 8–9% (Figure 6 and Supplementary Figure 7). Peptide effect was linear in the 0.04–4 nM range. At a concentration of 0.04 nM, IL-1β concentration in the pulled down fraction was 112% in comparison to the positive control (without any peptide inhibitor). Peptide 3.0 displayed the lowest IC_50_ value of 303.5 pM, which confirms that this inhibitor is the most potent of all the variants.

Peptide 4.2 was designed to test if the negative charges carried by Glu288—Asp289—Glu290 are essential for binding (Table 3). At a concentration of 40 nM, IL-1β concentration levels in the pulled down fraction dropped to 36% of the positive control (Figure 6 and Supplementary Figure 8). At the lowest concentration (0.04 nM), IL-1β concentration is 102% that of the positive control. Peptide 4.2 showed linearity in the 0.04–40 nM range. Though Peptide 4.2 does not show as strong of inhibitory capabilities as Peptide 3.0 at 40 nM, it is comparable to the original Peptide 1.0's inhibition at a lower peptide concentration.

At the highest concentration of 40 nM, Peptide 1.2 reduced IL-1β concentration to 35% of the positive control (Supplementary Figure 9). An increase in IL-1β concentration to 51 and 60% of the positive control was seen as the concentration of Peptide 1.2 decreased to 4 and 0.4 nM (Figure 6). Peptide 1.2 showed greater inhibition than Peptides 3.0 and 4.2 but was not as potent of an inhibitor as Peptide 3.0.

Finally, Peptide 2.0 (Table 3), which contains an alanine instead of arginine in position 286, was used as a negative control. It was predicted that without the arginine, the peptide should not be able to bind to the IL-1β:IL-1RI complex. At the highest concentration of 25 nM, the level IL-1β in the pulled-down fraction was 99% of the positive control (Figure 6 and Supplementary Figure 10). This shows that replacing the Arg286 with an alanine affects the peptide's ability to bind and inhibit IL-1β complex formation and is a further confirmation of the arginine 286 role.

### Peptide Variants 3.0 and 4.2 Are the Most Potent Inhibitors of Reporter Cell Line IL-1β Signaling Cascade

**Figure 7:**
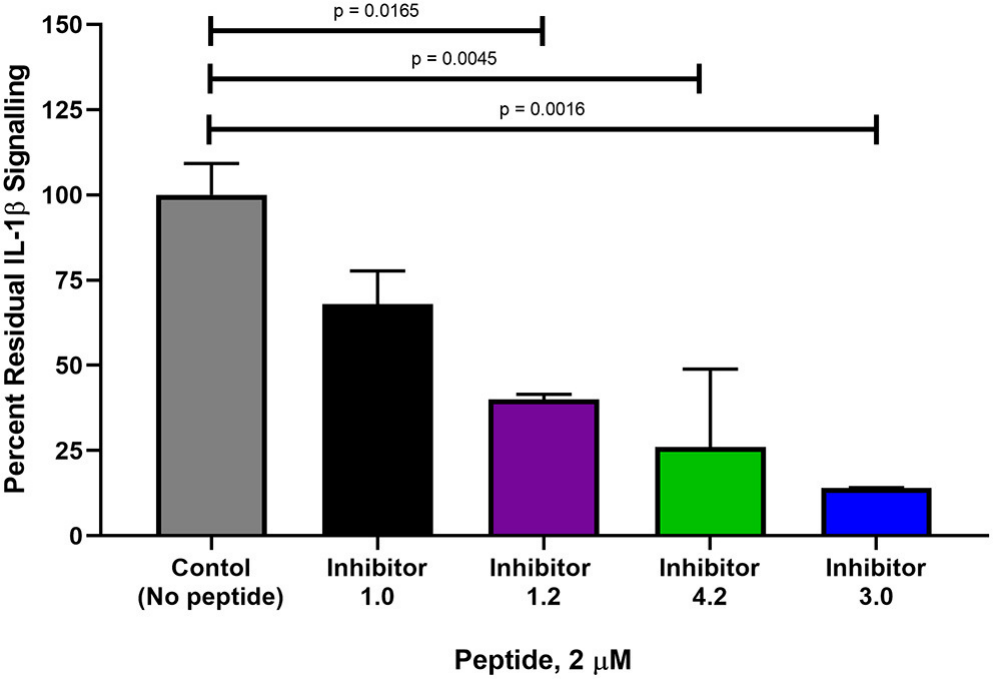
Comparison of percent interleukin signaling between peptide variants in HEK-Blue reporter cells. The amount of IL-1β downstream signaling was measured in NF-κB and AP-1 expression (AU) determined from reported SEAP levels. IL-1β stimulation was used as a positive control at a concentration of 2.5 ng and is set at 100% IL-1β signaling. Each peptide variant was then evaluated in triplicate and percentage of total signaling was calculated. The *p*-values were calculated with a 1-way Anova using Dunnett's test for multiple comparisons.

Of the 9 candidate peptides evaluated, Peptides 1.0, 1.2, 3.0, and 4.2 proved to be the most effective with inhibitory capabilities higher than 45% IL-1β pull down inhibition (Figure 6 and Supplementary Figures 6–14), and therefore were chosen for further analysis using the HEK-Blue IL-1β reporter cell assay. Both Peptides 3.0 and 4.2 displayed significantly greater inhibition than Peptide 1.0 at 2 μM (54% difference, *p* = 0.0016 and 42% difference, *p* = 0.0045, respectively, Figure 7). Peptide 1.2 was superior to Peptide 1.0 (28% difference, *p* = 0.0165 at 2 μM), but to a lesser extent than Peptides 3.0 and 4.2 (Figure 7). Therefore, the peptide variants 3.0 and 4.2 have proven their inhibitory capabilities in both *in vitro* experiments and are excellent candidates for future *in vivo* mouse model testing.

## Discussion

### IL-1β Complex Formation and IL-1RAcP Arg286 Essential Role

IL-1RAcP is essential for mediating IL-1β signaling. Interaction of IL-1β with the extracellular domains of IL-1RI and IL-1RAcP causes juxtaposition of the intracellular Toll-IL-1 receptor domains of IL-1RI and IL-1RAcP and triggers intracellular signaling (Figure 1A; Sims, 2010). The IL-1RI extracellular domain binds to IL-1β with nanomolar affinity, facilitating the subsequent binding of the receptor ligand complex to IL-1RAcP as a necessary step to initiate signaling (Figure 1B; Sims, 2010). IL-1β is a monomeric protein containing a 12 stranded beta-trefoil domain that does not change substantially after integration into the complex with the receptor and the accessory protein (Wang et al., 2010).

The extracellular portions of both IL-1RI and its family member IL-1RAcP contain three immunoglobulin-like domains (D1-D3), with IL-1RI wrapping around IL-1β such that domains D1, D2, and D3 all contribute to IL-1β binding. The IL-1β/IL-1RI binary complex recruits IL-1RAcP and remains virtually unchanged upon binding (Wang et al., 2010). IL-1RAcP uses the convex surface formed by the D2 and D3 domains to interact with the pre-formed ligand-receptor complex (Wang et al., 2010). The D1 domain of IL-1RAcP points away from IL-1β/IL-1RI and does not participate to the binding (Wang et al., 2010). A rotation of the IL-1RAcP D3 domain is necessary for it to touch the IL-1β/IL-1RI complex (Sims, 2010). Inhibition of the ternary complex formation blocks dimerization and hinders the SAPK/JNK signaling pathway. For these reasons, inhibitors that target IL-1RAcP or interfere with its ability to bind the IL-1β/IL-1R1 binary complex may represent a new paradigm in osteoarthritis treatment.

Prior protein painting experiments identified sequences on each separate protein in the complex that were all adjacent and juxtaposed to each other based on 3-D modeling (Vigers et al., 1997; Thomas et al., 2012). The highly conserved IL-1RAcP sequence that emerged indicated that the accessory protein, required for interleukin signaling, binds to the receptor-ligand complex in a single, very narrow region, and interacts at three points with the receptor and the ligand (Sims, 2010; Luchini et al., 2014). This evidence is fully supported by the crystallography data and provides new information that supports the hypothesis that rotation of the third immunoglobin domain in IL-1RAcP is necessary for it to touch the composite face of the receptor and ligand (Blech et al., 2013).

Using the results from the initial protein painting experiments (Luchini et al., 2014), a synthetic peptide inhibitor was developed mimicking the region from the IL-1RAcP that contains the Arg286 residue. This peptide abolished IL-1β signaling in a cell line model and inhibited complex formation in a pull-down assay. It was also shown that when the positively charged arginine is substituted with another amino acid (such as a less structurally complex, uncharged alanine residue), the inhibitory abilities of the peptide are eliminated. Also, since the Arg286 residue is conserved evolutionarily, it means that this region is of great importance in the binding of this hotspot (Thangudu et al., 2012). Hence, the Arg286 residue specifically must play an important role in IL-1β complex formation.

Not only have our prior results regarding the role of Arg286 (IL-1RAcP R306) as an energetically favorable hotspot for IL-1β complex binding been confirmed by crystallography and protein painting, it has been shown through alanine scanning that this hotspot is also significantly involved, energetically, in IL-1β binary complex formation (IL-1β:IL-1RI) (Luchini et al., 2014). Computational results in this work highlighted the stable, conserved interaction network of the complex in MD simulations (Supplementary Figure 4 and Supplementary Table 1) and the energetic influence of Arg286 (Supplementary Table 13), as well as other residue scaffolds on the IL-1β:IL-1RI scaffold that peptide mimetics should target. Fingerprint interactions reinforce the importance of Arg286 for protein interface and show general stability in the overall interactions that occur between the IL-1β, IL-1RI, and IL-1RAcP complex (Supplementary Tables 2–13). Moving forward, we have greater insights into the consistent interactions in this interface network, as well as transient interactions that were observed in simulation that can be further exploited in the future for inhibitor design.

Beyond IL-1β, IL-1RAcP is a secondary receptor necessary for downstream signaling of another cytokine, IL-33. Protein painting of these complexes has elucidated two distinct mechanisms for receptor engagement in IL-1β and IL-33 signaling. IL-33 was shown to bind to its cognate receptor (ST2) first in order to constrain the conformation of the receptor in a beneficial position to bind to IL-1RAcP (Günther et al., 2017). It was also shown that Arg286 is a unique single site hotspot for IL-1β:IL-1RI:IL-1RAcP, and is not involved with ternary complex formation of IL-33:ST2:IL-1RAcP (Günther et al., 2017). Hence, the Arg286 residue specifically must play an important role in solely IL-1β complex formation and continues to demonstrate its value as a therapeutic target to disrupt IL-1β signaling.

### Confirmation of Biological Significance of Arg286 Hot Spot via IL-1β Induced Inflammation Mouse Model and Therapeutic mAb Validation

Since the advent of the therapeutic mAb in 1986, the market for mAbs has increased dramatically to a global sales revenue of almost $75 billion as of 2013 (Ecker et al., 2015). mAbs are very popular with pharmaceutical companies due to their ability to be efficiently produced, high specificity, low immunogenicity, and low toxicity, which makes them safe therapeutic option for patients (Maggon, 2007). Also, immunogenicity can be further reduced by the humanization of mAbs derived from mice by replacing the V framework and constant regions with sequences from humans. Currently, using transgenic mice to derive human mAbs is a preferred route of production (Harding et al., 2010). Thus, it was postulated that a mAb targeting Arg286 could be a valid therapeutic strategy for OA. We used Peptide 1.0 to create two mouse monoclonal antibodies (7B8D7 and 6D3H5).

Antibodies were confirmed to form a stable complex with IL-1RAcP via pull down assays (Figure 3 and Supplementary Figure 5). Specific epitope binding was experimentally validated using protein painting (Figure 3). These results show that protein painting is a versatile technology that can be used not only to identify hot spots of interaction for rational drug design, but also to rapidly and effectively detect epitope binding of biologicals in physiological conditions.

A novel mouse model of inflammation via IL-1β subcutaneous injection in the ankle joint was proposed in this study. Different mouse models involving IL-1β knock-outs (Faggioni et al., 1998; Rider et al., 2011) or TNFα injections (Sharpe et al., 1988) have been used in the past to investigate inflammation. The mouse model proposed herein is ideal for assessing the inhibitory capabilities of the candidate peptide and antibody drugs for IL-1β complex disruption and inflammation suppression due to its lack of genetic alteration and mimicry of a PTA microenvironment with the least amount of suffering for the mice. Ly6G+/IL-1RAcP+ local granulocyte recruitment was considered as measure of inflammation (Granstein et al., 1986; Wesche et al., 1997; Seifer et al., 2008; Miller, 2011; Turner et al., 2014). Since it is known that symptoms of inflammation (such as swelling, pain, redness, etc.) are due to the effects of granulocyte recruitment, it is pertinent to stain the tissues for the presence or absence of these cells in the afflicted tissues (Turner et al., 2014).

Mouse tissues were also used to validate the affinity and specificity of the 7B8D7 mAb toward the IL-1RAcP using immunohistochemistry staining. Both 7B8D7 and 6D3H5 mAbs induced a dramatic decrease in the number of migrated granulocytes in comparison to the untreated, stimulated control. At a concentration of 3 and 1.34 nM, the 7B8D7 mAb was able to inhibit the induced inflammation by 81 and 78% in the ankle joint of the mouse model, showing a dose response effect (Figure 5). Collectively, *in vitro* and *in vivo* data reported in this study confirm that the Arg286 hot spot plays a significant role in IL-1β induced inflammation and is a valid therapeutic target for OA and PTA.

### Peptide Variants Validate Arg286 Hot Spot as Potential Therapeutic Target

During this study, it was shown that the peptide variants 3.0 and 4.2 were able to inhibit the IL-1β signaling cascade to a much higher degree than the original Peptide 1.0 at their most effective concentrations in both the *in vitro* receptor complex immunoprecipitation assay and interleukin signaling cell culture model. The key variations from the leader sequence that had an impact on this signaling cascade are specific amino acid substitutions for cyclization, changes in charge distribution, and secondary interaction strengthening (substituting methionine for threonine, for example).

Peptide cyclization has been previously shown to dramatically improve peptide drug performance (Hu et al., 2004; Choi and Joo, 2020; Jing and Jin, 2020; Sohrabi et al., 2020), and more than 40 cyclic peptides have been approved for use in the clinic over the past decades (Jing and Jin, 2020). Cyclic peptides have distinct advantages over their acyclic counterparts targeting the same region: (1) greater binding affinity and selectivity toward target molecules due to their increased rigidity that reduces the entropy term in the Gibbs free energy equation, (2) resistance to enzymatic degradation because their rigid structure might prevent peptidases from accessing their cleavage site, and (3) increased membrane permeability in some cases (Choi and Joo, 2020). In this study, two of the best performing peptides, Peptides 1.2 and 4.2, were cyclic peptides.

Ring size can exert a great influence on a cyclized peptide's activity as demonstrated in this study by the larger inhibitory activity that Peptide 4.2 has over 4.0 (Supplementary Figures 8, 12) on the reduction of key inflammatory signaling cascade components. It has been shown previously that depending on the target region, there is a tendency toward increased potency with larger ring size during peptide formation due to increased selectivity and decreased degradation by proteases (Andrews et al., 2004). Larger cyclized peptides tend to be more effective on cell surface receptors than intracellular proteins due to issues with cell membrane penetration (Driggers et al., 2008). But, when ring size gets too large (more than 20 membered rings), then therapeutic activity will begin to decrease (Hu et al., 2004). In the immunoprecipitation model, Peptide 4.2 (Supplementary Figure 8) displayed improved inhibitory capabilities when compared to Peptide 4.1 (Supplementary Figure 13) and Peptide 4.3 (Supplementary Figure 14), which have shorter cycle lengths. Therefore, it is reasonable to deduce that the optimum ring to disrupt the IL-1β complex formation is an 18-member ring.

Having a larger ring size is only one factor in the design of Peptide 4.2, which was obtained by substituting Gln for Glu and Asn for Asp. Peptide 4.2 outperformed the lead Peptide 1.0 in the immunoprecipitation and cell culture models. In the cell line model (Figure 7), Peptide 4.2 had a better performance than Peptide 1.2, which also had an 18-member ring size but retained its Glu and Asp. It has been shown that enhanced charge distribution can have a great influence on the potency of a therapeutic peptide, even larger effect than a shift in hydrophobicity (Feder et al., 2000). One can deduce that the effect of a larger ring size and a change in charge distribution both contributed to the potency of the peptide.

In the immunoprecipitation experiments, very low concentrations of Peptides 3.0 and 4.2 (100 pM) caused a small percentage increase of complex concentrations with respect to the absence of peptide inhibitor. This evidence makes critical the need to ensure sustained delivery of the peptide to the target site in future *in vivo* applications. We envision intra-articular injection of the antagonist to block the vicious inflammatory cycle initiated by joint injury, which is at the basis of osteoarthritis development. A number of strategies have been proposed in the literature to achieve sustained and extended drug availability of intra-articular drugs (Rai and Pham, 2018), such as (1) drug incorporation in amphiphilic polymeric micelles that self-assemble into nanostructure, and (2) drug encapsulation in hydrogel microparticles with very low immunogenicity [e.g., poly(lactic-co-glycolic acid), PLGA, in the Zilretta formulation of triamcinolone acetonide approved by the FDA to manage osteoarthritis knee pain].

Surprisingly, the substitution for methionine in the 293 position of the leader peptide sequence exhibited an even greater inhibitory effect than variant 4.2. This mutation was performed to better occupy the gap in interaction space between Ala305 and Tyr307 of IL-1R1 at the peptide interface. Originally, Thr293 had transient backbone interactions with Ala305 and 306 in the crystal structure. To better occupy this space with a favorable interaction, methionine was chosen as a larger hydrophobic, but polarizable residue to interact with the IL-1R1 peptide sequence. Thr293 was selected rather than Thr291 given the observed and needed interactions of Thr291 with Arg208. The distance between the closest two atoms of the secondary IL-1R1 Arg208 and the IL-1RAcP Thr291 is 2.8, 3.8, and 7.6 Å, respectively, in dominant morphologies from MD simulation, highlighting both the need for an interaction at this position with the movement of Thr291 toward Arg208. Thr293 was mostly an underutilized residue at the IL-1R1- IL-1RAcP interface. It has been shown in different TNF-ligand complexes that methionine plays an important role in protein complex stabilization through interactions with aromatic groups on neighboring residues by its sulfur atom (Valley et al., 2012). Although the mechanism is not quite understood, this stabilization could be due to the extreme hydrophobic nature of methionine (Brosnan and Brosnan, 2006). Also, methionine's sulfur atom's enormous polarizability produces strong non-polar interaction, which add to the strength of binding (Gellman, 1991). Given the presence of Tyr307 and His291 of IL-1R1 in a loop region of the crystal structure position of Thr293 of IL-1RAcP, methionine was selected to be both a larger, more bulky amino acid to better fill the void in this interaction space, as well as have favorable qualities to interact with aromatic residues in this region of IL-1RI. All these characteristics could have contributed to the success of Peptide 3.0 in inhibiting IL-1β signaling complex formation. Further investigation into substituting methionine for other residues in the Peptide 1.0 sequence should be completed to fully study its effect IL-1β complex disruption.

The results of this study indicate that peptides with larger cyclized ring structures and substitutions with charge distribution contributing stronger electrostatic interactions tend to be more potent inhibitors of IL-1β complex formation and downstream signaling. Also, substitution in a secondary interaction point with methionine dramatically increases potency of the Peptide 1.0 inhibitor. Peptides 4.2 and 3.0 have proven their *in vitro* capabilities and are excellent candidates for future *in vivo* testing.

It is to be noted that, while antibodies 6D3H5 and 7B8D7 target IL-1RAcP, peptide inhibitors target IL-1β. Target protein concentration has important implications on the stoichiometry of the inhibitor for *in vivo* applications. IL-1β is present at the site of joint injury at much higher concentrations than IL-1RAcP; thus, an IL-1β inhibitor will require higher concentrations than an inhibitor targeting IL-1RAcP, given equivalent affinity. However, in face of this stoichiometric disadvantage, peptides inhibitors tested in this study showed high potency, with IC_50_ values in the low hundreds of picomolar range; moreover, delivery systems can be pursued in the future to afford a sustained and prolonged peptide availability *in vivo*.

In conclusion, computational and experimental approaches at the molecular, cellular and animal model level validated Arg286 of IL-1RAcP as a novel and effective drug target for IL-1β induces inflammation in OA. Computational studies confirmed that Arg286 is the highest energy mediator of binding and a dominant contributor to the interaction network of the ternary complex. Additionally, computational studies highlighted other residues at the interface for further probing, as well as highlighted rationale for mutational design and efficacy of inhibitors presented here. In an optimized mouse model of osteoarthritis, monoclonal antibodies targeting Arg286 significantly reduced the number of granulocyte infiltrating the site of joint injury. Peptides mimicking the Arg286 region effectively inhibited IL-1β complex formation and IL-1β signaling in pull down systems and cell line assays. Peptide cyclization and multivalency enhancement induced optimal results. Overall, the disruption of the interface network involving the Arg286 region of IL-1RAcP is a promising target of next generation treatments for osteoarthritis.

## Data Availability Statement

The original contributions presented in the study are publicly available. This data can be found here: MassIVE MSV000086480.

## Ethics Statement

The animal study was reviewed and approved by Institutional Animal Care and Use Committee, Office of Research Integrity and Assurance, George Mason University.

## Author Contributions

AD designed the study, performed the experiments, and wrote the manuscript. AL and LL designed the study and wrote the manuscript. VE assisted with study design and manuscript review. KM performed cell-based assay experiments. NV performed antibody characterization experiments. RJ, KL, CG, MP, and AB performed the molecular dynamics and quantum mechanics calculations and simulations. AH assisted with figure creation and manuscript writing. MP designed and illustrated peptide variants. All authors contributed to the article and approved the submitted version.

## Conflict of Interest

VE, AHS, LL, and AL are inventors on patents related to the protein painting. Monet Pharmaceuticals licensed the rights of these patents that are owned by George Mason University. AHS, LL, and AL own shares of Monet Pharmaceuticals. The remaining authors declare that the research was conducted in the absence of any commercial or financial relationships that could be construed as a potential conflict of interest.

## Footnotes

^1^*Office of Research Computing Site – ARGO – Hardware Specs*. http://wiki.orc.gmu.edu/index.php/About_ARGO (accessed January 11, 2021)
