# Supplementary material for: Characterization and Validation of Arg286 Residue of IL-1RAcP as a Potential Drug Target for Osteoarthritis
Source: Front Chem. 2021 Feb 3;8:601477. doi: 10.3389/fchem.2020.601477 (PMC7886681; doi:10.3389/fchem.2020.601477)
Supplement: Supplementary file 1 [file Table_1.doc]

# ***Supplementary Information***

# ***Characterization and Validation of Arg286 residue of IL-1RAcP as a Potential Drug Target for Osteoarthritis***

**Angela Dailing^1^, Kelsey Mitchell^1^, Ngoc Vuong^1^, Kyung Hyeon Lee^2^, Reva Joshi^1^, Virginia Espina^1^, Amanda Haymond Still^1^, Carter J. Gottschalk^3^, Anne M. Brown^3,4^, Mikell Paige^2^, Lance A. Liotta^1^, and Alessandra Luchini^1^**

**Table of contents:**

- **Supplementary Figure S1**. Root-mean-square deviation (RMSD) of backbone atoms of the IL-1β - IL-1RI- IL-1RAcP complex. ***2***
- **Supplementary Figure S2**. Radius of gyration of the IL-1β - IL-1RI - IL-1RAcP complex. ***3***
- **Supplementary Figure S3**. Root-mean-square fluctuation (RMSF) of backbone atoms for the IL-1β - IL-1RI - IL-1RAcP complex. ***4***
- **Supplementary Figure S4.** Dominant morphology of the IL-1 β - IL-1RI - IL-1RAcP complex from backbone RMSD clustering of the over the last 20 ns of simulation time. ***5***
- **Supplementary Figure S5.** 6D3H5 Monoclonal Antibody forms a stable complex with IL-1RAcP and Targets Arg286. ***6***
- **Supplementary Figure S6.** Peptide 1.0 inhibition of IL-1β ligand and receptor complex using His-tagged IL-1RAcP pull-down assay. ***7***
- **Supplementary Figure S7**. Peptide 3.0 variant inhibition of IL-1β ligand and receptor complex using His-tagged IL-1RAcP pull-down assay. ***8***
- **Supplementary Figure S8**. Peptide 4.2 variant inhibition of IL-1β ligand and receptor complex using His-tagged IL-1RAcP pull-down assay. ***9***
- **Supplementary Figure S9**. Peptide 1.2 variant inhibition of IL-1β ligand and receptor complex using His-tagged IL-1RAcP pull-down assay. ***10***
- **Supplementary Figure S10.** Peptide 2.0 variant inhibition of IL-1β ligand and receptor complex using His-tagged IL-1RAcP pull-down assay. ***11***
- **Supplementary Figure S11.** Peptide 1.1 inhibition of IL-1β ligand and receptor complex using His-tagged IL-1RAcP pull-down assay. ***12***
- **Supplementary Figure S12**. Peptide 4.0 inhibition of IL-1β ligand and receptor complex using His-tagged IL-1RAcP pull-down assay. ***13***
- **Supplementary Figure S13.** Peptide 4.1 inhibition of IL-1β ligand and receptor complex using His-tagged IL-1RAcP pull-down assay. ***14***
- **Supplementary Figure S14**. Peptide 4.3 inhibition of IL-1β ligand and receptor complex using His-tagged IL-1RAcP pull-down assay. ***15***
- **Supplementary Table S1**. Key residues at the IL-1β - IL-1RI - IL-1RAcP interface. ***16***
- **Supplementary Table S2**. Fingerprint interaction analysis for IL-1β, replicate 1. ***17***
- **Supplementary Table S3**. Fingerprint interaction analysis for IL-1R1, replicate 1. ***18***
- **Supplementary Table S4**. Fingerprint interaction analysis for IL-1RAcP, replicate 1. ***19***
- **Supplementary Table S5**. Fingerprint interaction analysis for IL-1β, replicate 2. ***20***
- **Supplementary Table S6.** Fingerprint interaction analysis for IL-1R1, replicate 2. ***21***
- **Supplementary Table S7**. Fingerprint interaction analysis for IL-1RAcP, replicate 2. ***22***
- **Supplementary Table S8.** Fingerprint interaction analysis for IL-1β, replicate 3. ***23***
- **Supplementary Table S9.** Fingerprint interaction analysis for IL-1R1, replicate 3. ***24***
- **Supplementary Table S10**. Fingerprint interaction analysis for IL-1RAcP, replicate 3. ***25***
- **Supplementary Table S11.** Fingerprint interaction analysis for IL-1β, starting structure. ***26***
- **Supplementary Table S12.** Fingerprint interaction analysis for IL-1R1, starting structure. ***27***
- **Supplementary Table S13**. Fingerprint interaction analysis for IL-1RAcP, starting structure. ***28***
- **Supplementary Table S14**. MM-GBSA per residue energy decomposition of the ternary complex. ***29***


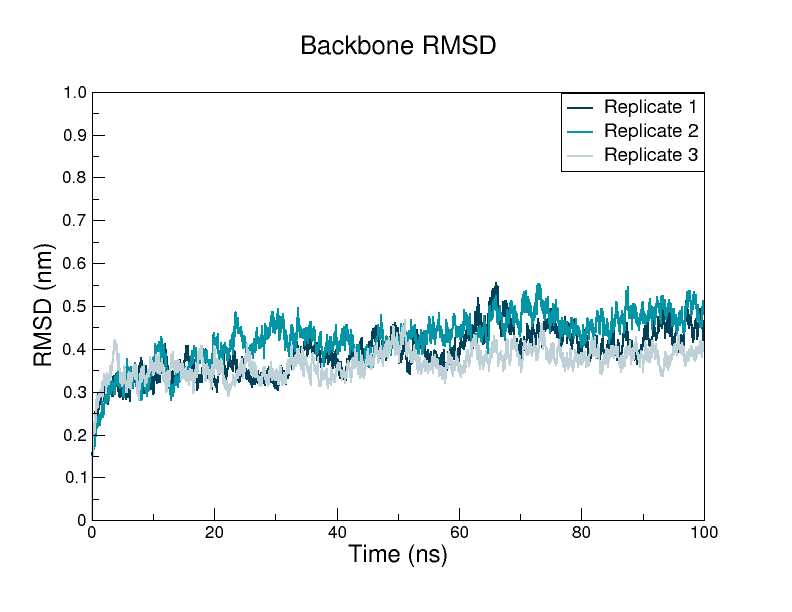


**Supplementary Figure S1. Root-mean-square deviation (RMSD) of backbone atoms of the IL-1β - IL-1RI- IL-1RAcP complex**. RMSD is shown for each replicate and compares the current frame to the starting structure.


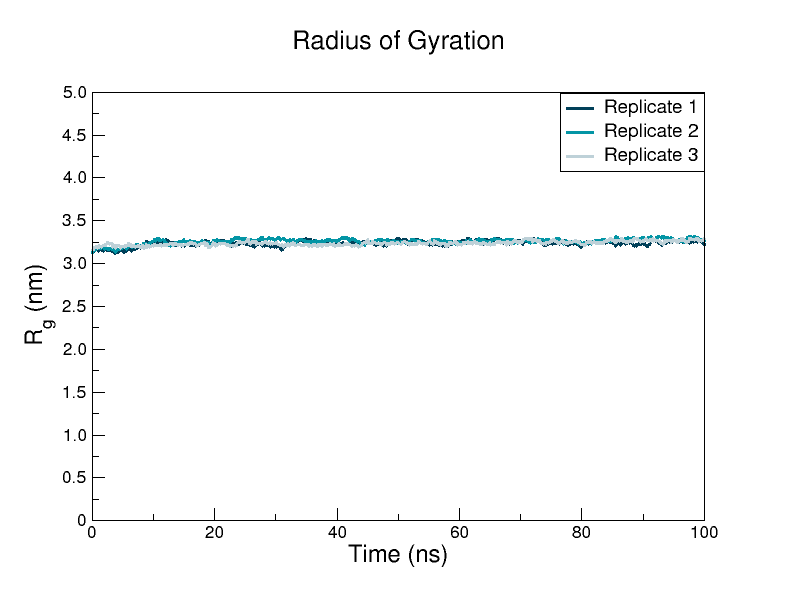


**Supplementary Figure S2. Radius of gyration of the IL-1β - IL-1RI - IL-1RAcP complex.** Radius of gyration is shown for each replicate as a complex over time.


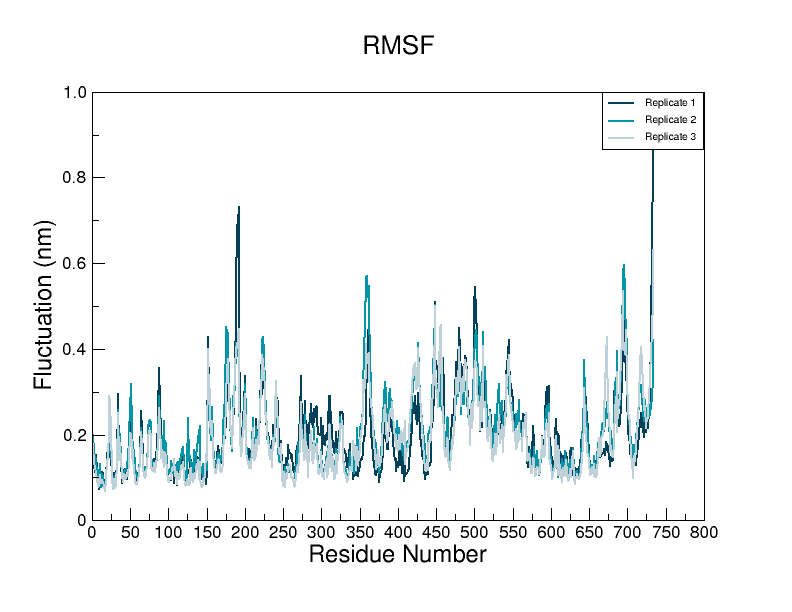


**Supplementary Figure S3. Root-mean-square fluctuation (RMSF) of backbone atoms for the IL-1β - IL-1RI - IL-1RAcP complex.** RMSF is shown per residue for each replicate over the duration of the simulation. Residues are numbered continuously in order of subunit -- IL-1β - IL-1R1 - IL-1RAcP.


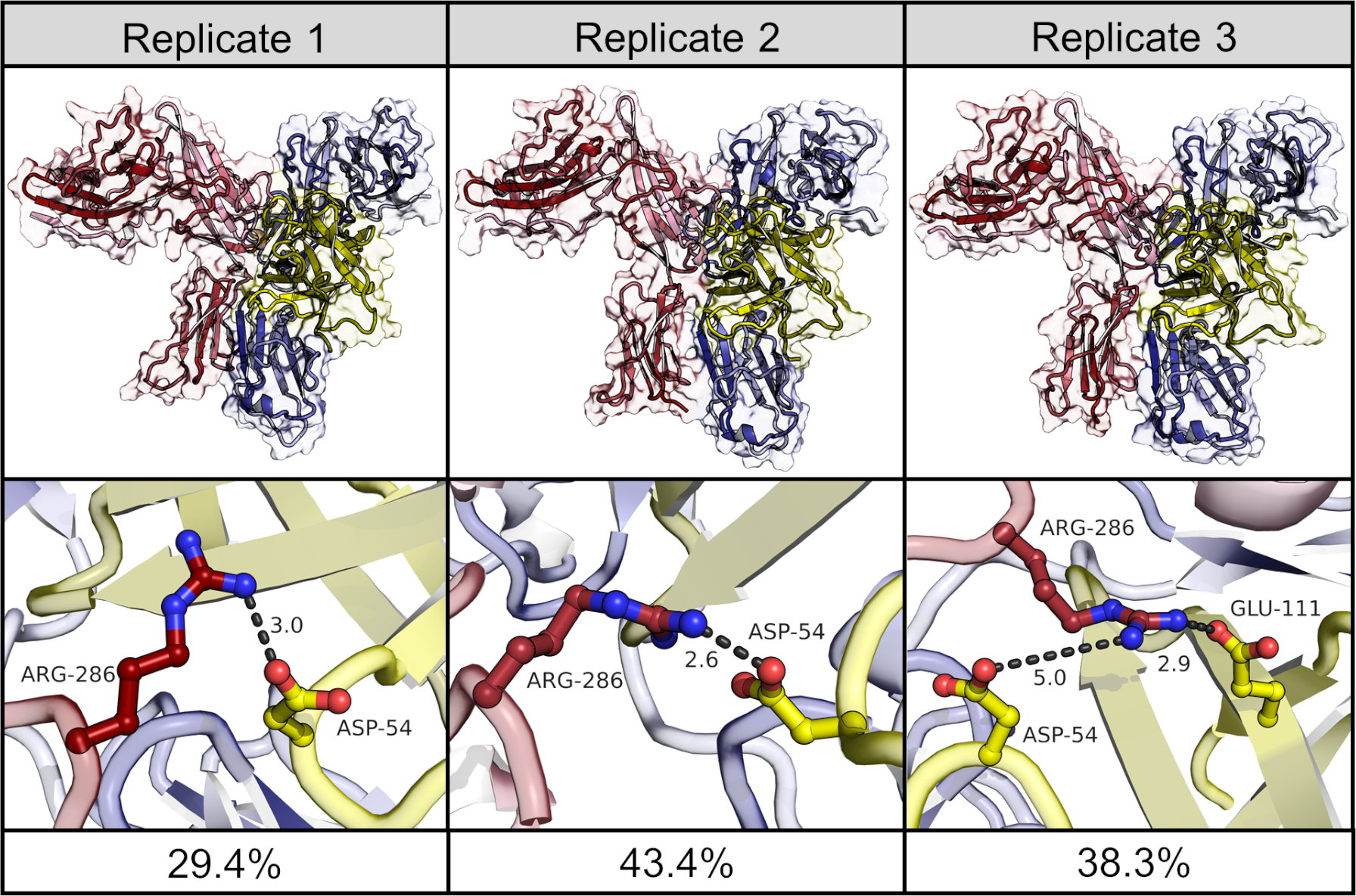


**Supplementary Figure S4. Dominant morphology of the IL-1 β - IL-1RI - IL-1RAcP complex from backbone RMSD clustering of the over the last 20 ns of simulation time.** The most populated cluster was selected for each replicate and percentage of frames representing that structure are shown. Complex is shown as cartoon, which each subunit colored yellow -- IL-1β, blue -- IL-1R1, red -- IL-1RAcP. RMSD calculations between the most populated cluster and second most populated cluster was less than 0.3 nm.


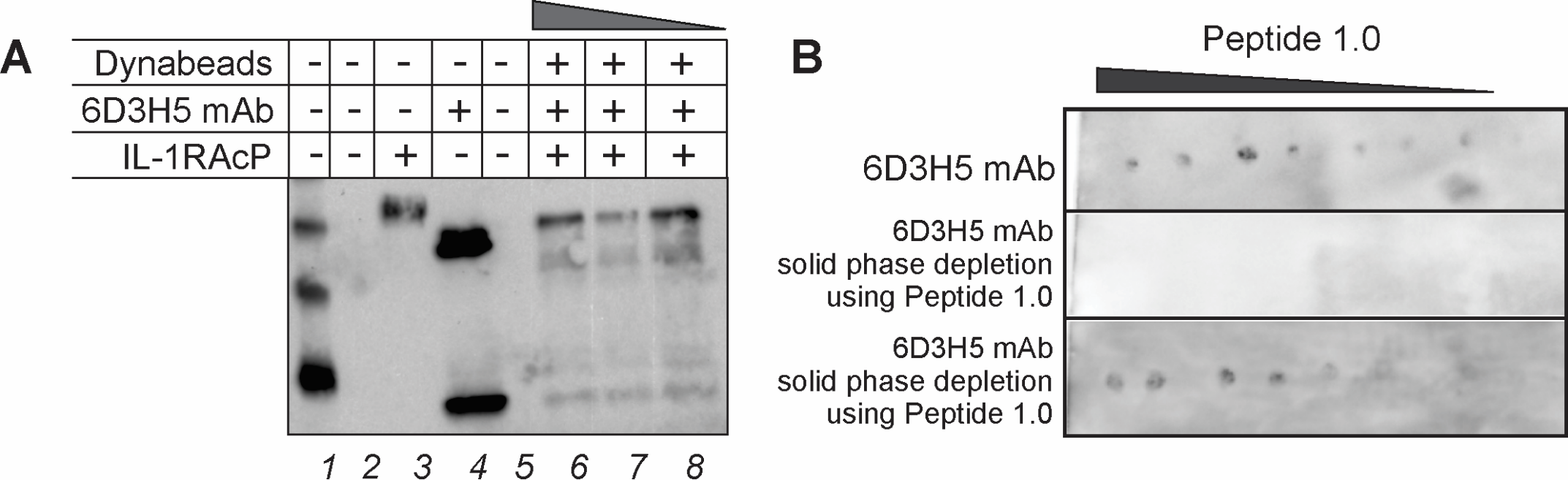


**Supplementary Figure S5. 6D3H5 Monoclonal Antibody forms a stable complex with IL-1RAcP and Targets Arg286.** A.) 6D3H5 monoclonal antibody (mAb) coupled Dynabeads were incubated with IL-1RAcP at various concentrations and eluted by boiling in sample buffer. Lane 1 is the molecular ladder, lane 2 and 5 are empty, lane 3 contains IL-1RAcP without beads, and lane 4 contains 6D3H5 mAb without beads. Lane 6 to 8 contain the elution from the antibody-coupled beads incubated with 80 ng, 60 ng, and 40 ng of IL-1RAcP, respectively. B) Dot blot and solid phase affinity depletion show that Arg286 is the epitope of the 6D3H5 mAb. Peptide 1.0 was spotted on a nitrocellulose membrane (2 μg, 1 μg, 0.5 μg, 0.25 μg, and 0 μg). 6D3H5 diluted 1:1000 in blocking solution (5% dry milk dissolved in PBS additioned with 0.5% Tween) was allowed to incubate with the membrane for 60 minutes at room temperature. Signal was obtained using a horse radish peroxidase coupled anti-mouse secondary antibody, and luminol as substrate. Further confirmation that 6D3H5 is an anti-Arg286 antibody was obtained via solid phase affinity depletion conducted as described in [doi: 10.1186/s12967-015-0701-z]. Peptides 1.0 and 2.0 (100 μg) were used to coat ELISA plate wells. The wells were then washed with PBS three times and incubated with 5 μg of 6D3H5 mAb for 2 hours at 37 °C. After incubation the antibody supernatant was recovered used to stain the dot blot containing Peptide 1.0. No immunoreactivity in the mAb preparation after immunodepletion with Peptide 1.0 was observed, while the immunoreactivity was maintained after immunodepletion with Peptide 2.0.


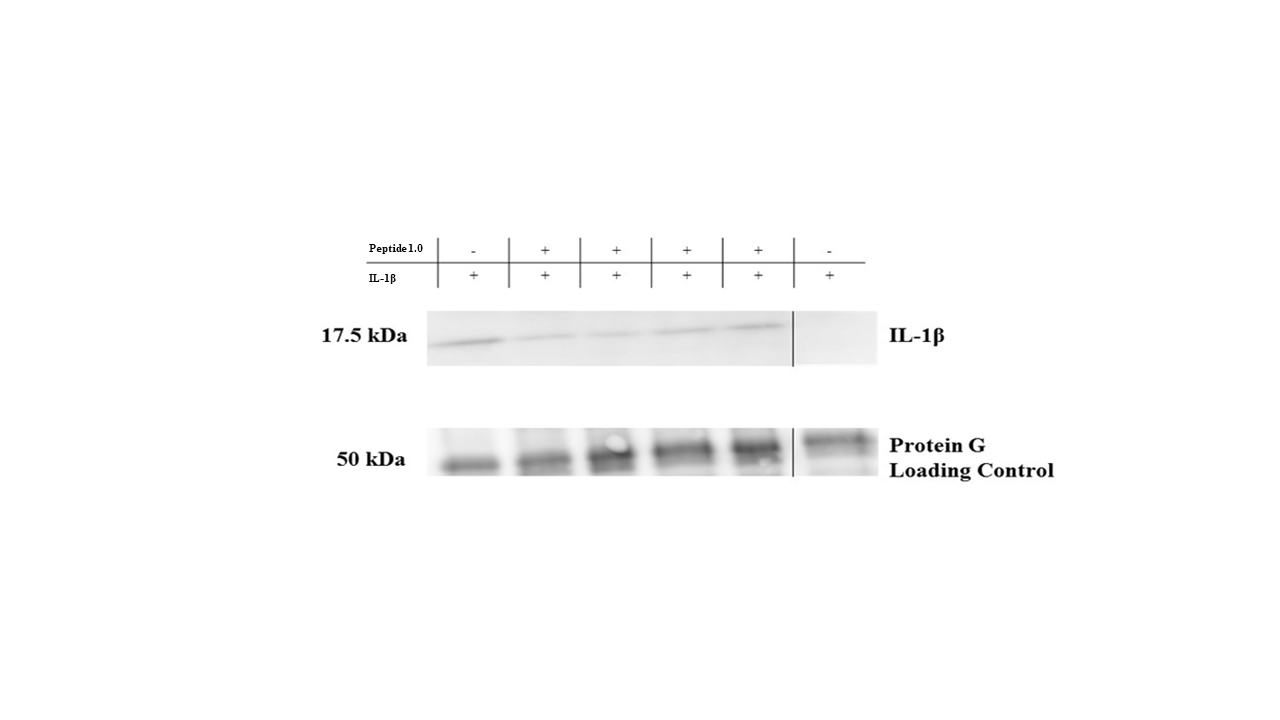


1 2 3 4 5 6

**Supplementary Figure S6. Peptide 1.0 inhibition of IL-1β ligand and receptor complex using His-tagged IL-1RAcP pull-down assay.** Concentrations of IL-1β, IL-1RI, and IL-1RAcP are as follows: 40 ng, 100 ng, and 40 ng. Peptide 1.0 concentrations used are as follows in descending concentration: 124, 62, 31, and 12.5 nM. Lane 1 is the positive control, Lanes 2-5 are the protein complexes incubated with the peptide variant in decreasing concentrations, and Lane 6 is the negative control. The positive control is the ternary IL-1β complex without any inhibitor and the negative control is IL-1β alone with the beads. The bottom panel displays the protein G loading control for each corresponding lane.


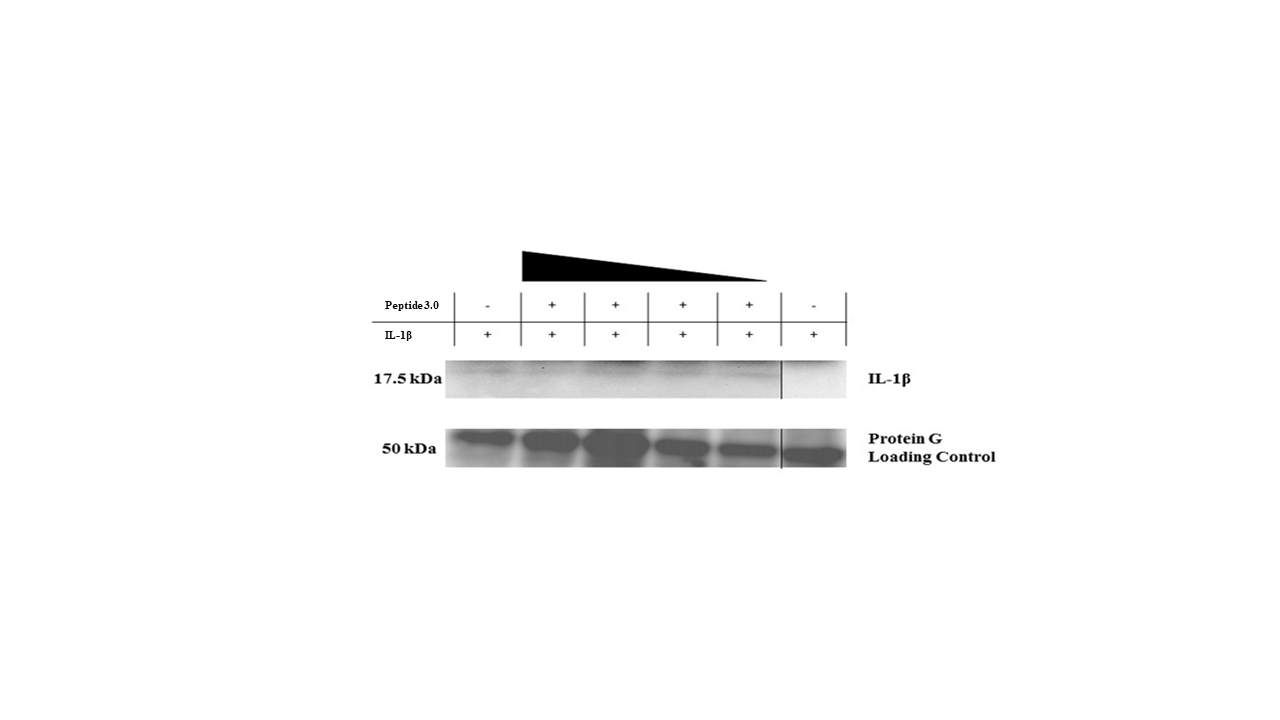


1 2 3 4 5 6

**Supplementary Figure S7. Peptide 3.0 variant inhibition of IL-1β ligand and receptor complex using His-tagged IL-1RAcP pull-down assay.** Concentrations of IL-1β, IL-1RI, and IL-1RAcP are as follows: 40 ng, 100 ng, and 40 ng. Peptide 3.0 concentrations used are as follows in descending concentration: 40, 4, 0.4, and 0.04 nM. Lane 1 is the positive control, Lanes 2-5 are the protein complexes incubated with the peptide variant in decreasing concentrations, and Lane 6 is the negative control. The positive control is the ternary IL-1β complex without any inhibitor and the negative control is IL-1β alone with the beads. The bottom panel displays the protein G loading control for each corresponding lane.


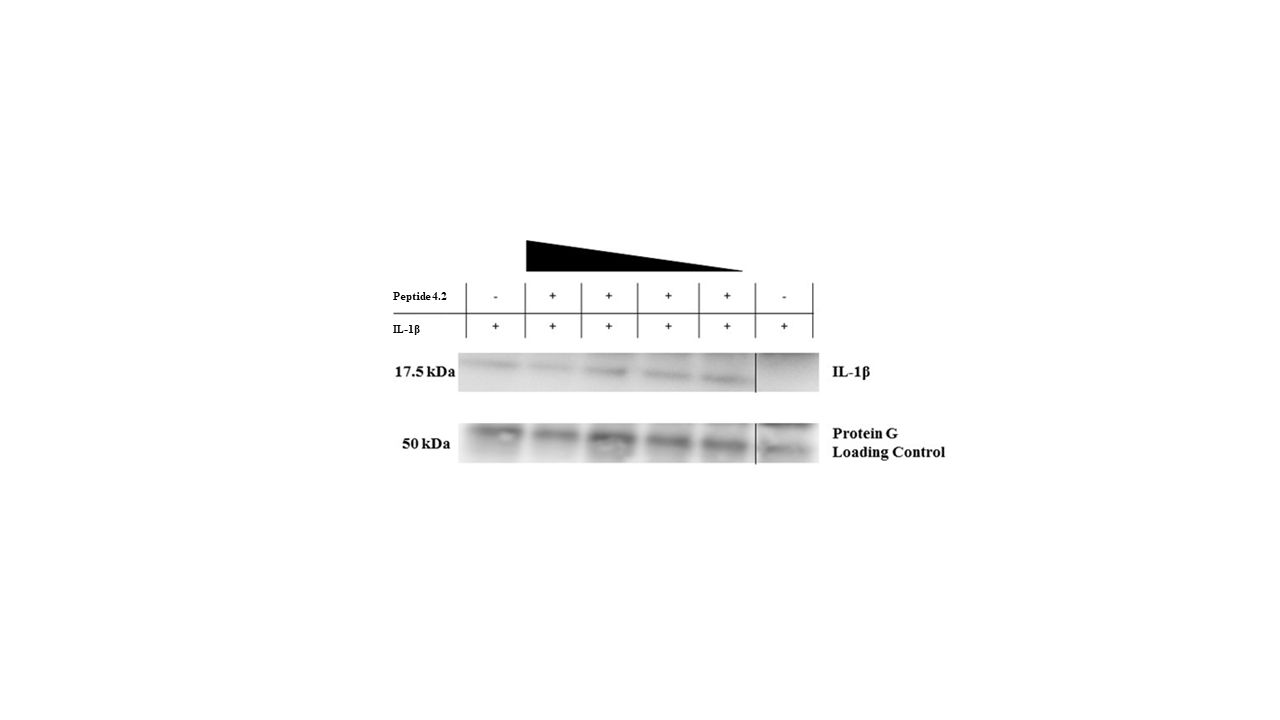


1 2 3 4 5 6

**Supplementary Figure S8. Peptide 4.2 inhibition of IL-1β ligand and receptor complex using His-tagged IL-1RAcP pull-down assay.** Concentrations of IL-1β, IL-1RI, and IL-1RAcP are as follows: 40 ng, 100 ng, and 40 ng. Peptide 4.2 concentrations used are as follows in descending concentration: 40, 4, 0.4, and 0.04 nM. Lane 1 is the positive control, Lanes 2-5 are the protein complexes incubated with the peptide variant in decreasing concentrations, and Lane 6 is the negative control. The positive control is the ternary IL-1β complex without any inhibitor and the negative control is IL-1β alone with the beads. The bottom panel displays the protein G loading control for each corresponding lane.


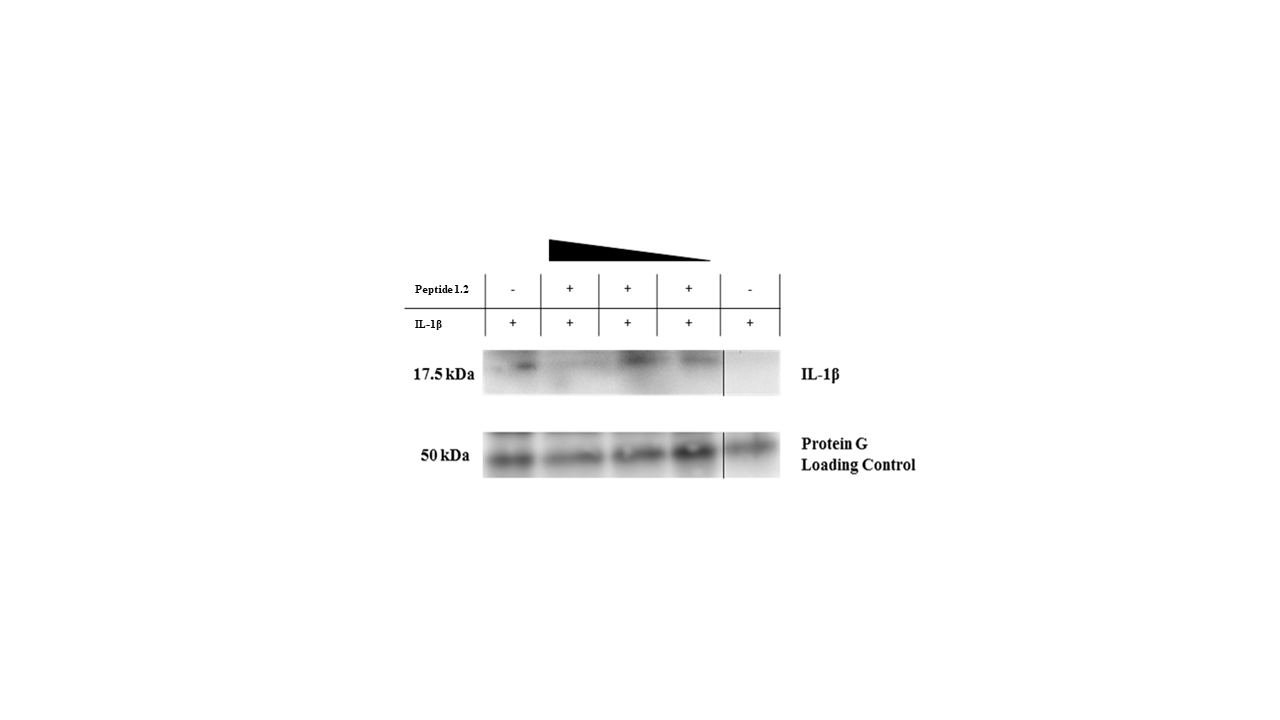


1 2 3 4 5

**Supplementary Figure S9.** **Peptide 1.2 inhibition of IL-1β ligand and receptor complex using His-tagged IL-1RAcP pull-down assay.** Concentrations of IL-1β, IL-1RI, and IL-1RAcP are as follows: 40 ng, 100 ng, and 40 ng. Peptide 1.2 concentrations used are as follows in descending concentration: 40, 4, 0.4, and 0.04 nM. Lane 1 is the positive control, Lanes 2-4 are the protein complexes incubated with the peptide variant in decreasing concentrations, and Lane 5 is the negative control. The positive control is the ternary IL-1β complex without any inhibitor and the negative control is IL-1β alone with the beads. The bottom panel displays the protein G loading control for each corresponding lane.


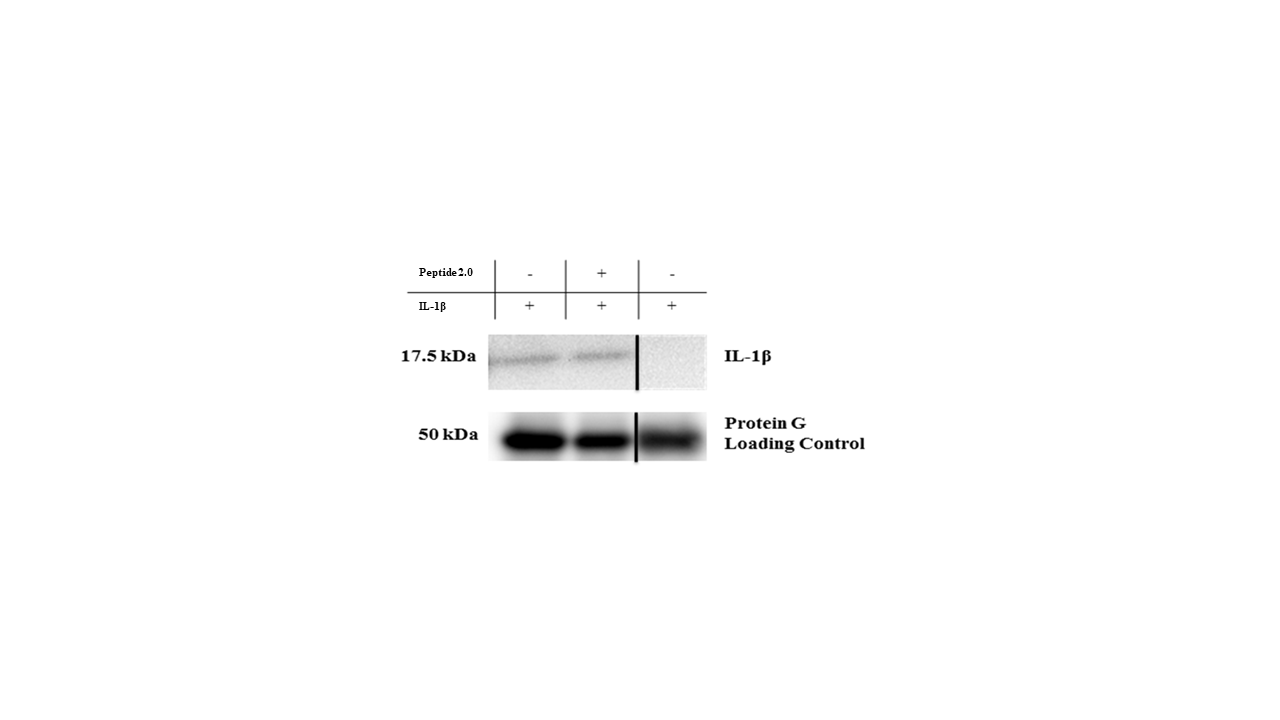


1 2 3

**Supplementary Figure S10. Peptide 2.0 inhibition of IL-1β ligand and receptor complex using His-tagged IL-1RAcP pull-down assay.** Concentrations of IL-1β, IL-1RI, and IL-1RAcP are as follows: 40 ng, 100 ng, and 40 ng. Peptide 2.0 concentration used was 25 nM. Lane 1 is the positive control, Lanes 2 is the protein complex incubated with Peptide 2.0, and Lane 3 is the negative control. The positive control is the ternary IL-1β complex without any inhibitor and the negative control is IL-1β alone with the beads. The bottom panel displays the protein G loading control for each corresponding lane.


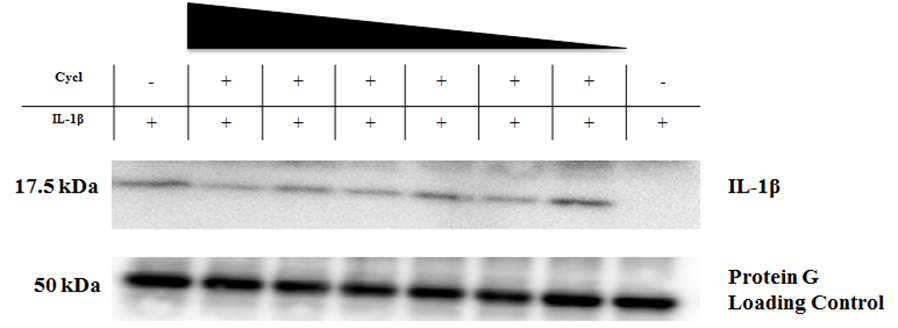


1 2 3 4 5 6 7 8

Supplementary Figure S11. Peptide 1.1 inhibition of IL-1β ligand and receptor complex using His-tagged IL-1RAcP pull-down assay. Concentrations of IL-1β, IL-1RI, and IL-1RAcP are as follows: 40 ng, 100 ng, and 60 ng. Peptide 1.1 concentrations used are as follows in descending concentration: 25, 12.3, 8.2, 6.1, 5, and 2.5 nM. Lane 1 is the positive control, Lanes 2-7 are the protein complexes incubated with the peptide variant in decreasing concentrations, and Lane 8 is the negative control. The bottom panel displays the protein G loading control for each corresponding lane.


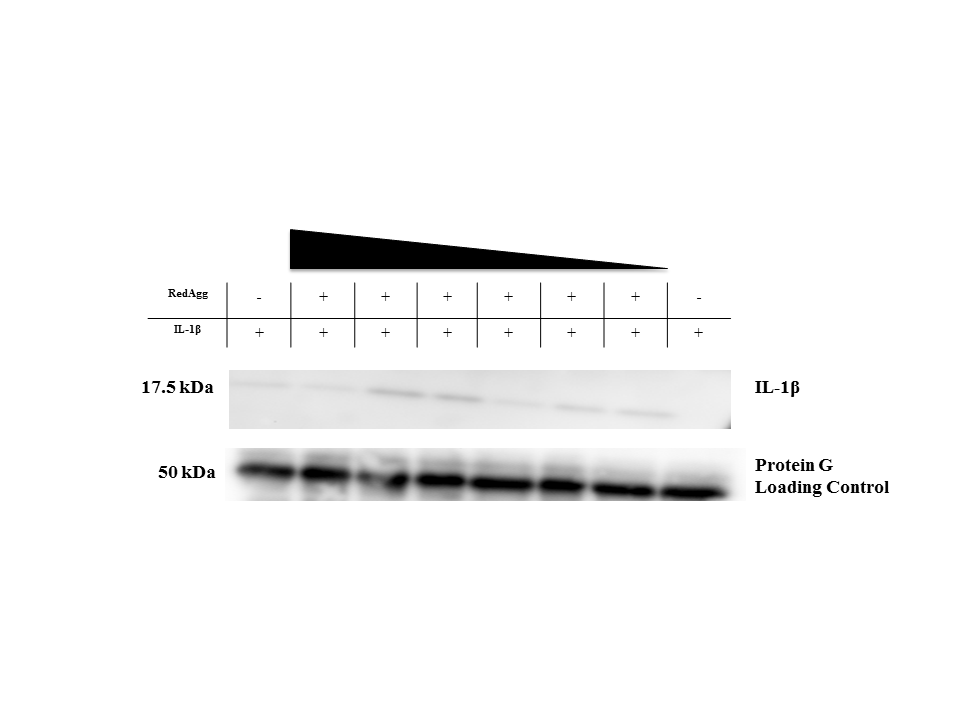


1 2 3 4 5 6 7 8

Supplementary Figure S12. Peptide 4.0 inhibition of IL-1β ligand and receptor complex using His-tagged IL-1RAcP pull-down assay. Concentrations of IL-1β, IL-1RI, and IL-1RAcP are as follows: 40 ng, 100 ng, and 60 ng. Peptide 4.0 concentrations used are as follows in descending concentration: 25, 12.4, 8.3, 6.2, 5, and 2.5 nM. Lane 1 is the positive control, Lanes 2-7 are the protein complexes incubated with the peptide variant in decreasing concentrations, and Lane 8 is the negative control. The bottom panel displays the protein G loading control for each corresponding lane.


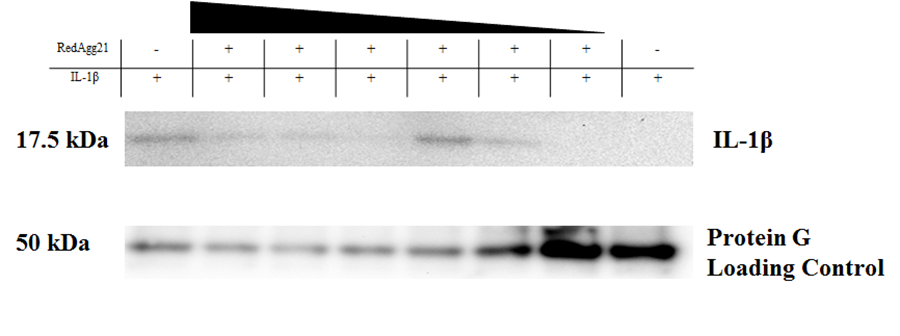


1 2 3 4 5 6 7 8

Supplementary Figure S13. Peptide 4.1 inhibition of IL-1β ligand and receptor complex using His-tagged IL-1RAcP pull-down assay. Concentrations of IL-1β, IL-1RI, and IL-1RAcP are as follows: 40 ng, 100 ng, and 60 ng. Peptide 4.1 concentrations used are as follows in descending concentration: 41.8, 20.8, 14, 10.4, 8.4, and 2.4 nM. Lane 1 is the positive control, Lanes 2-7 are the protein complexes incubated with the peptide variant in decreasing concentrations, and Lane 8 is the negative control. The bottom panel displays the protein G loading control for each corresponding lane.


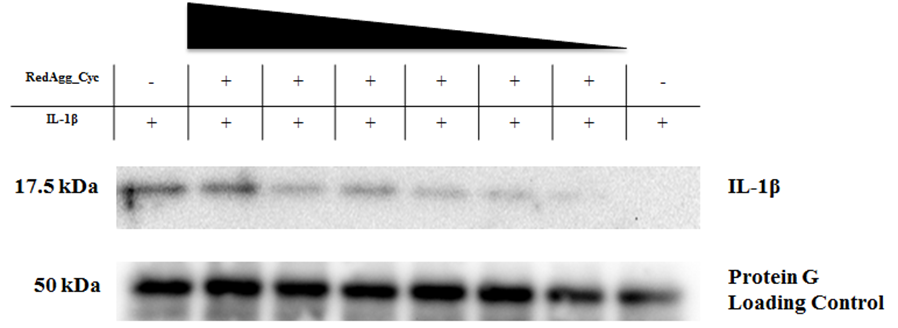


1 2 3 4 5 6 7 8

Supplementary Figure S14. Peptide 4.3 inhibition of IL-1β ligand and receptor complex using His-tagged IL-1RAcP pull-down assay. Concentrations of IL-1β, IL-1RI, and IL-1RAcP are as follows: 40 ng, 100 ng, and 60 ng. Peptide 4.3 concentrations used are as follows in descending concentration: 25, 13, 8.7, 6.5, 5.2, and 2.6 nM. Lane 1 is the positive control, Lanes 2-7 are the protein complexes incubated with the peptide variant in decreasing concentrations, and Lane 8 is the negative control. The bottom panel displays the protein G loading control for each corresponding lane.

**Supplementary Table S1**. **Key residues at the IL-1β - IL-1RI - IL-1RAcP interface.** Interactions that were observed in dominant morphologies and across replicates of simulation. Interactions were identified by Schrodinger Maestro fingerprint analysis.

| IL-1RAcP | Average Distance (Å) | Prevalence Across Replicates | IL-1β | |
| --- | --- | --- | --- | --- |
| Hydrogen Bonds | | | | |
| GLN165 | 2.9 | 66% | GLN141 | |
| ASN168 | 3.1 | 100% | GLY140 | |
| SER185 | 2.7 | 100% | ASP145 | |
| Salt Bridges | | | | |
| GLU132 | 2.9 | 66% | LYS109 | |
| ARG286 | 3.5 | 100% | ASP54 | |
| ARG286* | 2.9 | 33% | GLU111 | |
| Aromatic | | | | |
| PHE167 | 3.9 | 100% | GLN141 | |
| IL-1RAcP | Average Distance (Å) | Prevalence | | IL-1RI |
| Hydrogen Bonds | | | | |
| HIS226 | 3.0 | 66% | | ASP304 |
| Polar | | | | |
| THR291 | 3.4 | 66% | | ARG208 |

* Indicates the interaction with GLU111 which was observed in one replicate. Glu111 is in close proximity of ASP54 to suggest it may play a role in creating a negatively charged binding site for IL-1RIRAcP: 286

**Supplementary Table S2. Fingerprint interaction analysis for IL-1β.** Residues shown of IL-1β were determined to have an interaction with any residues of IL-1R1 or IL-1RAcP. Data shown are for the dominant morphology of replicate 1. Interactions were determined by Schrodinger Maestro.

| Interaction Type | Backbone | Polar | Hydro-phobic | Hydrogen Bond Acceptor | Hydrogen Bond Donor | Aromatic | Charged |
| --- | --- | --- | --- | --- | --- | --- | --- |
| Residues | ALA1 ARG4 SER13 GLN14 HIS30 LEU31 GLN32 GLY33 GLU50 GLU51 SER52 ASN53 LYS55 GLU105 ASN107 LYS109 LEU110 ASN129 LYS138 GLY139 GLY140 GLN141 ASP142 PHE150 VAL151 | ARG4 ARG11 GLN15 LYS27 HIS30 GLN32 GLN48 GLU51 ASN53 ASP54 LYS93 LYS94 LYS103 GLU105 ASN107 ASN108 LYS109 GLU111 GLN126 GLU128 ASN129 GLN141 THR144 ASP145 GLN149 | ALA1 LEU29 MET36 PHE46  ILE56  ILE104 ILE106 PRO131 ILE143 PHE150 | ALA1 GLN15 LEU31 GLN32 GLU50 GLU51 SER52 ASP54 GLU105 GLY140 ASP145 | ALA1 ARG4 GLN15 GLN32 GLY33 LYS93 LYS94 ASN107 ASN108 LYS109 GLN141 | PHE46 PHE150 | ARG4 ARG11 LYS27 GLU51 ASP54 LYS93 LYS94 LYS103 GLU105 LYS109 GLU111 GLU128 ASP145 |

**Supplementary Table S3. Fingerprint interaction analysis for IL-1R1.** Residues shown of IL-1R1 were determined to have an interaction with any residues of IL-1β or IL-1RAcP. Data shown are for the dominant morphology of replicate 1. Interactions were determined by Schrodinger Maestro.

| Interaction Type | Backbone | Polar | Hydrophobic | Hydrogen Bond Acceptor | Hydrogen Bond Donor | Aromatic | Charged |
| --- | --- | --- | --- | --- | --- | --- | --- |
| Residues | LEU15 VAL16 ALA109 ILE110 PHE111 LYS112 GLN113 LYS114 GLY119 GLY122 PRO126 TYR127 GLU202 GLU203 ASN204 LYS205 PRO206 THR207 ARG208 ILE211 VAL212 ALA215 LEU237 SER238 ASP239 ILE240 GLY247 ILE250 TYR261 TYR262 LYS298 ASN299 THR300 HIS301 | LYS112 GLN113 LYS114 ASP120 ARG163 ASN168 GLU202 ASN204 LYS205 THR207 ARG208 SER238 LYS244 ASP251 GLU259 ASP260 SER263 ASN266 LYS270 ARG271 ARG272 LYS298 THR300 HIS301 | ILE13  LEU15 VAL16 PRO26 LEU29 ILE110 PHE111 LEU115 VAL124 PRO126 TYR127 PHE130 VAL160 ILE165 ILE199 LEU201 PRO206 VAL210 ILE240 VAL249 TYR261 LEU275 PHE296 ILE303 | VAL16 ALA109 ILE110 LYS114 ASN168 GLU203 ASN204 ILE211 LEU237 ASP239 ILE250 ASP251 GLU259 ASP260 TYR261 | VAL16 LYS112 LYS114 LYS244 TYR261 ASN266 ARG272 LYS298 THR300 | PHE111 TYR127 PHE130 TYR261 PHE296 | LYS112 LYS114 ASP120 ARG163 GLU202 LYS205 ARG208 LYS244 ASP251 GLU259 ASP260 LYS270 ARG271 ARG272 LYS298 |

**Supplementary Table S4. Fingerprint interaction analysis for IL-1RAcP**. Residues shown of IL-1RAcP were determined to have an interaction with any residues of IL-1β or IL-1R1. Data shown are for the dominant morphology of replicate 1. Interactions were determined by Schrodinger Maestro.

| Interaction Type | Backbone | Polar | Hydrophobic | Hydrogen Bond Acceptor | Hydrogen Bond Donor | Aromatic | Charged |
| --- | --- | --- | --- | --- | --- | --- | --- |
| Residues | GLU132 GLN165 ASN166 PHE167 ASN168 SER185 GLY215 ASN219 THR287 LYS326 | GLU132 ASN166 ASN168 SER185 LYS218 ASN219 HIS226 THR247 SER285 ARG286 THR287 ASP289 THR291 | ILE131 MET159 PHE167 VAL170 LEU180 ILE181 LEU183 ILE184 VAL221 ILE244 TYR249 | GLU132 GLN165 LYS326 | ASN168 SER185 LYS218 HIS226 ARG286 | PHE167 TYR249 | GLU132 LYS218 ARG286 ASP289 |

**Supplementary Table S5. Fingerprint interaction analysis for IL-1β.** Residues shown of IL-1β were determined to have an interaction with any residues of IL-1R1 or IL-1RAcP. Data shown are for the dominant morphology of replicate 2. Interactions were determined by Schrodinger Maestro.

| Interaction Type | Backbone | Polar | Hydrophobic | Hydrogen Bond Acceptor | Hydrogen Bond Donor | Aromatic | Charged |
| --- | --- | --- | --- | --- | --- | --- | --- |
| Residues | ALA1 PRO2 SER13 GLN14 MET20 SER21 GLY22 LEU31 GLN32 GLY33 GLU50 GLU51 SER52 ASN53 ASP54 LYS55 GLU105 ASN107 PRO131 GLY139 GLY140 VAL151 | ARG4 ARG11 SER13 GLN14 GLN15 SER21 GLU25 LYS27 HIS30 GLN32 GLN34 GLN38 GLU51 ASP54 LYS93 LYS94 LYS103 GLU105 ASN107 ASN108 LYS109 GLN126 ASN129 LYS138 GLN141 ASP145 GLN149 | PRO2  LEU6 PRO23 LEU29 LEU31 PHE46 ILE56  ILE104 ILE106 MET130 PRO131 ILE143 PHE150 | SER13 GLN15 MET20 GLU25 GLN34 GLU51 SER52 ASP54 LYS55 GLU105 ASN107 GLY140 ASP145 GLN149 VAL151 | ARG4 GLN15 SER21 GLN32 GLY33 LYS93 LYS94 LYS103 ASN107 GLN126 GLY139 | PHE46 PHE150 | ARG4 ARG11 GLU25 LYS27 GLU51 ASP54 LYS93 LYS94 LYS103 GLU105 LYS109 LYS138 ASP145 |

**Supplementary Table S6. Fingerprint interaction analysis for IL-1R1.** Residues shown of IL-1R1 were determined to have an interaction with any residues of IL-1β or IL-1RAcP. Data shown are for the dominant morphology of replicate 2. Interactions were determined by Schrodinger Maestro.

| Interaction Type | Backbone | Polar | Hydro-phobic | Hydrogen Bond Acceptor | Hydrogen Bond Donor | Aromatic | Charged |
| --- | --- | --- | --- | --- | --- | --- | --- |
| Residues | GLU10 GLU11 LYS12 VAL16 PRO28 GLN108 ALA109 ILE110 PHE111 LYS112 GLN113 LYS114 ASP120 GLY121 PRO126 GLU203 ASN204 LYS205 ALA215 ASN216 LEU237 SER238 ASP239 ILE240 GLU259 ASP260 LYS270 ARG271 LYS298 ASN299 THR300 ASP304 ALA305 | ARG9 GLU11 LYS12 ASN30 GLN108 LYS112 GLN113 LYS114 ASP120 ASN154 ARG163 GLU202 ASN204 ARG208 ASN216 GLU217 GLN236 ASP239 LYS244 GLU252 GLU259 ASP260 LYS270 ARG271 THR294 LYS298 THR300 HIS301 ASP304 | ILE13 LEU15 VAL16 PRO28 PRO31 ALA109 PHE111 VAL124 PRO126 TYR127 VAL160 ILE165 MET167 PRO206 LEU237 ILE240 TYR242 TYR261 LEU275 PHE296 TYR307 | GLU11 ALA109 ILE110 LYS114 ALA215 GLU217 LEU237 ASP239 GLU252 GLU259 ASP304 | ARG9 LYS12 GLN108 LYS114 ARG163 ASN204 GLN236 TYR242 LYS244 LYS270 LYS298 THR300 HIS301 TYR307 | PHE111 TYR127 TYR242 TYR261 PHE296 TYR307 | ARG9 GLU11 LYS12 LYS112 LYS114 ASP120 ARG163 GLU202 ARG208 GLU217 ASP239 LYS244 GLU252 GLU259 ASP260 LYS270 ARG271 LYS298 ASP304 |

**Supplementary Table S7. Fingerprint interaction analysis for IL-1RAcP.** Residues shown of IL-1RAcP were determined to have an interaction with any residues of IL-1β or IL-1R1. Data shown are for the dominant morphology of replicate 2. Interactions were determined by Schrodinger Maestro.

| Interaction Type | Backbone | Polar | Hydro-phobic | Hydrogen Bond Acceptor | Hydrogen Bond Donor | Aromatic | Charged |
| --- | --- | --- | --- | --- | --- | --- | --- |
| Residues | GLY134 ASN166 PHE167 ASN168 ASN169 SER185 LYS326 | GLU132 ARG137 ASN168 ASN169 SER185 ASN219 HIS226 ARG286 LYS326 | ILE131 ILE135 MET159 TYR162 PHE167 ILE171 ILE181 LEU183 ILE184 VAL224 ILE244 TYR249 | GLU132 ASN168 SER185 ASN219 LYS326 | ASN168 ASN169 SER185 ASN219 HIS226 ARG286 LYS326 | TYR162 PHE167 TYR249 | GLU132 ARG137 ARG286 LYS326 |

**Supplementary Table S8. Fingerprint interaction analysis for IL-1β.** Residues shown of IL-1β were determined to have an interaction with any residues of IL-1R1 or IL-1RAcP. Data shown are for the dominant morphology of replicate 3. Interactions were determined by Schrodinger Maestro.

| Interaction Type | Backbone | Polar | Hydro-phobic | Hydrogen Bond Acceptor | Hydrogen Bond Donor | Aromatic | Charged |
| --- | --- | --- | --- | --- | --- | --- | --- |
| Residues | ALA1 PRO2 VAL3 ARG4 GLN14 HIS30 LEU31 GLN32 GLY33 GLN34 SER52 ASN53 ASP54 LYS55 LYS92 LYS93 LYS94 ILE104 GLU105 ASN107 GLY139 GLY140 GLN141 | ARG4 ARG11 GLN14 GLN15 HIS30 GLN32 GLN34 GLN48 ASP54 LYS92 LYS93 LYS103 GLU105 ASN107 ASN108 LYS109 GLU111 GLN126 GLU128 ASN129 THR137 GLN141 ASP145 GLN149 | ALA1  LEU6  LEU29 MET36 PHE46  ILE56  ILE104 ILE106 ALA127 ILE143 MET148 PHE150 | ALA1 GLN14 GLN15 GLN32 ASP54 GLU105 GLU111 GLU128 ASN129 GLY140 ASP145 | ALA1 ARG4 ARG11 GLN15 GLN32 GLY33 GLN34 GLN48 LYS93 LYS94 LYS103 GLU105 LYS109 GLN126 GLN141 | PHE46 PHE150 | ARG4 ARG11 ASP54 LYS92 LYS93 LYS103 GLU105 LYS109 GLU111 GLU128 ASP145 |

**Supplementary Table S9. Fingerprint interaction analysis for IL-1R1.** Residues shown of IL-1R1 were determined to have an interaction with any residues of IL-1β or IL-1RAcP. Data shown are for the dominant morphology of replicate 3. Interactions were determined by Schrodinger Maestro.

| Interaction Type | Backbone | Polar | Hydro-  phobic | Hydrogen Bond Acceptor | Hydrogen Bond Donor | Aromatic | Charged |
| --- | --- | --- | --- | --- | --- | --- | --- |
| Residues | LEU15 VAL16 GLN108 ALA109 ILE110 PHE111 LYS112 GLN113 LYS114 ALA118 ASP120 GLY121 GLY122 PRO126 TYR127 ASN204 LYS205 PRO206 LEU237 SER238 ASP239 ILE240 ILE250 ASP260 TYR261 TYR262 LYS298 ASN299 THR300 | GLN108 GLN113 LYS114 ASP120 GLU129 ASN154 SER158 ARG163 ASN204 ARG208 ASN216 GLN236 SER238 GLU252 GLU259 ASP260 LYS270 LYS298 THR300 HIS301 ASP304 | ILE13  LEU15 VAL16 PRO26 PHE111 LEU115 LEU123 VAL124 PRO126 TYR127 VAL160 ILE165 LEU201 PRO206 LEU237 ILE240 VAL249 TYR261 LEU275 | VAL16 GLN108 ALA109 ILE110 LYS112 LYS114 ASP120 GLY122 LEU237 SER238 ASP239 ILE250 GLU252 GLU259 ASP260 TYR261 THR300 ASP304 | VAL16 LYS114 TYR127 ARG163 ARG208 GLN236 TYR261 THR300 HIS301 | PHE111 TYR127 TYR261 | LYS114 ASP120 GLU129 ARG163 ARG208 GLU252 GLU259 ASP260 LYS270 LYS298 ASP304 |

**Supplementary Table S10. Fingerprint interaction analysis for IL-1RAcP.** Residues shown of IL-1RAcP were determined to have an interaction with any residues of IL-1β or IL-1R1. Data shown are for the dominant morphology of replicate 3. Interactions were determined by Schrodinger Maestro.

| Interaction Type | Backbone | Polar | Hydro-phobic | Hydrogen Bond Acceptor | Hydrogen Bond Donor | Aromatic | Charged |
| --- | --- | --- | --- | --- | --- | --- | --- |
| Residues | GLU132 TYR133 GLY134 GLN165 ASN166 PHE167 ASN168 SER185 | GLU132 GLN165 ASN168 SER185 LYS218 ASN219 HIS226 ARG286 THR291 | ILE131 ILE135 TYR162 PHE167 ILE171 LEU180 ILE181 LEU183 ILE184 ILE244 TYR249 | GLU132 GLN165 ASN166 ASN168 TYR249 THR291 | GLY134 ASN168 SER185 HIS226 ARG286 | TYR162 PHE167 TYR249 | GLU132 LYS218 ARG286 |

**Supplementary Table S11. Fingerprint interaction analysis for IL-1β.** Residues shown of IL-1β were determined to have an interaction with any residues of IL-1R1 or IL-1RAcP. Data shown are for the starting structure. Interactions were determined by Schrodinger Maestro.

| Interaction Type | Backbone | Polar | Hydro-  phobic | Hydrogen Bond Acceptor | Hydrogen Bond Donor | Aromatic | Charged |
| --- | --- | --- | --- | --- | --- | --- | --- |
| Residues | ALA1 PRO2 VAL3 ARG4 GLN14 SER21 HIS30 LEU31 GLN32 GLY33 SER52 ASN53 ASP54 LYS55 LYS92 LYS93 LYS94 ILE104 GLU105 ILE106 ASN107 ALA127 GLU128 LYS138 GLY139 GLY140 GLN141 | ARG4 ARG11 GLN14 GLN15 SER21 GLU25 LYS27 HIS30 GLN32 GLN34 ASP35 GLN48 GLU51 ASP54 LYS92 LYS93 LYS94 LYS103 GLU105 ASN107 ASN108 LYS109 GLU111 GLN126 GLU128 ASN129 LYS138 GLN141 THR144 ASP145 GLN149 | PRO2  LEU6  LEU29 PHE46  ILE56  ILE104 ILE106 ALA127 PRO131 ILE143 MET148 PHE150 | ALA1 GLN14 GLN15 SER21 GLU25 GLN32 GLY33 ASP35 GLU51 SER52 ASP54 GLU105 ASN129 GLY140 ASP145 | ALA1 ARG4 ARG11 GLN15 LYS27 GLN32 GLY33 GLN48 LYS93 LYS103 ASN107 ASN108 LYS109 GLN126 GLU128 ASN129 GLN141 | PHE46 PHE150 | ARG4 ARG11 GLU25 LYS27 ASP35 GLU51 ASP54 LYS92 LYS93 LYS94 LYS103 GLU105 LYS109 GLU111 GLU128 LYS138 ASP145 |

**Supplementary Table S12. Fingerprint interaction analysis for IL-1R1**. Residues shown of IL-1R1 were determined to have an interaction with any residues of IL-1β or IL-1RAcP. Data shown are for the starting structure. Interactions were determined by Schrodinger Maestro.

| Interaction Type | Backbone | Polar | Hydro-phobic | Hydrogen Bond Acceptor | Hydrogen Bond Donor | Aromatic | Charged |
| --- | --- | --- | --- | --- | --- | --- | --- |
| Residues | ILE13 ILE14 LEU15 VAL16 PRO28 LEU29 ASN30 GLN108 ALA109 ILE110 PHE111 LYS112 GLN113 LYS114 ALA118 GLY119 ASP120 GLY121 GLY122 VAL124 PRO126 GLU203 ASN204 LYS205 PRO206 LEU237 SER238 ASP239 ILE240 ILE250 ASP251 GLU259 ASP260 TYR261 LYS298 ASN299 THR300 HIS301 | ARG9 GLU11 ASN30 GLN108 LYS112 GLN113 LYS114 ASP120 GLU129 LYS132 SER158 LYS161 ARG163 ASN168 ASN204 LYS205 ARG208 GLN236 SER238 LYS244 ASP251 GLU252 GLU259 ASP260 SER263 LYS270 ARG271 LYS298 ASN299 THR300 HIS301 | ILE13  LEU15 VAL16 PRO31 PHE111 LEU115 VAL124 PRO126 TYR127 ILE165 MET167 PRO206 LEU237 ILE240 TYR242 VAL249 ILE250 TYR261 LEU275 | GLU11 VAL16 PRO28 LEU29 ALA109 ILE110 LYS112 LYS114 ALA118 ASP120 GLY122 GLU129 ASN204 SER238 ASP239 ILE250 ASP251 GLU252 GLU259 ASP260 | ARG9 ILE14 VAL16 ASN30 LYS112 LYS114 TYR127 LYS132 ARG163 ASN204 GLN236 SER238 LYS244 TYR261 LYS298 THR300 | PHE111 TYR127 TYR242 TYR261 | ARG9 GLU11 LYS112 LYS114 ASP120 GLU129 LYS132 LYS161 ARG163 LYS205 ARG208 LYS244 ASP251 GLU252 GLU259 ASP260 LYS270 ARG271 LYS298 |

**Supplementary Table S13. Fingerprint interaction analysis for IL-1RAcP.** Residues shown of IL-1RAcP were determined to have an interaction with any residues of IL-1β or IL-1R1. Data shown are for starting structure. Interactions were determined by Schrodinger Maestro.

| Interaction Type | Backbone | Polar | Hydro-phobic | Hydrogen Bond Acceptor | Hydrogen Bond Donor | Aromatic | Charged |
| --- | --- | --- | --- | --- | --- | --- | --- |
| Residues | GLU132 TYR133 GLY134 GLN165 ASN166 PHE167 ASN168 SER185 | GLU132 ASN166 ASN168 SER185 LYS218 ASN219 ARG286 THR291 | ILE131 ILE135 PHE167 ILE171 ILE181 LEU183 ILE184 TYR249 | GLU132 GLN165 ASN166 ASN168 | GLY134 ASN168 SER185 LYS218 ARG286 | PHE167 TYR249 | GLU132 LYS218 ARG286 |

**Supplementary Table S14. MM-GBSA per residue energy decomposition of the ternary complex.** Energies (kcal/mol) are shown as an average and standard deviation across all three replicates. Per replicate energies were calculated as an average

|  |  | MM-GBSA Total Energy Decomposition (kcal/mol) | | | | | |
| --- | --- | --- | --- | --- | --- | --- | --- |
| Subunit | Residue Number | Internal | VDW | Electrostatic | Polar Solvation | Non-polar solv. | Total |
| IL-1β | GLN14 | 32.3 ± 1.1 | -10.0 ± 1.0 | -82.4 ± 1.7 | 2.8 ± 0.6 | 0.1 ± 0.0 | -57.3 ± 1.1 |
| IL-1β | GLN126 | 23.6 ± 1.3 | -8.4 ± 0.6 | -72.1 ± 3.8 | -1.6 ± 2.4 | 0.1 ± 0.0 | -58.4 ± 2.2 |
| IL-1β | GLY140 | 78.6 ± 1.4 | -5.1 ± 0.3 | -74.4 ± 3.8 | -2.0 ± 1.7 | 0.6 ± 0.1 | -2.8 ± 1.2 |
| IL-1β | GLN141 | 29.4 ± 0.7 | -7.3 ± 1.4 | -67.7 ± 2.8 | -10.3 ± 3.0 | 0.2 ± 0.3 | -55.3 ± 1.9 |
| IL-1β | ASP142 | 103.6 ± 2.7 | -7.7 ± 0.3 | -102.1 ± 19.6 | -46.2 ± 15.5 | 0.0 ± 0.2 | -52.1 ± 1.4 |
| IL-1β | ILE143 | 94.4 ± 1.1 | -10.8 ± 0.1 | -75.4 ± 1.3 | -1.1 ± 0.5 | 0.3 ± 0.0 | 7.0 ± 0.7 |
| IL-1R1 | VAL160 | 63.6 ± 1.8 | -5.9 ± 0.8 | -58.0 ± 0.5 | -1.7 ± 0.8 | 0.2 ± 0.1 | -1.7 ± 0.8 |
| IL-1R1 | LYS161 | 119.4 ± 2.2 | -8.9 ± 0.4 | -102.1 ± 13.4 | -10.5 ± 14.9 | 0.0 ± 0.0 | -2.0 ± 1.0 |
| IL-1R1 | ASP162 | 103.6 ± 1.1 | -6.5 ± 0.8 | -154.3 ± 9.7 | -3.5 ± 10.5 | 0.1 ± 0.0 | -60.6 ± 1.3 |
| IL-1R1 | ARG163 | -187.2 ± 0.5 | -13.1 ± 0.1 | 30.2 ± 5.8 | 2.1 ± 7.5 | 0.0 ± 0.0 | -168.0 ± 1.2 |
| IL-1R1 | LEU164 | 80.4 ± 1.3 | -11.3 ± 0.1 | -85.2 ± 0.7 | -0.5 ± 0.1 | 0.0 ± 0.0 | -16.6 ± 0.7 |
| IL-1R1 | ILE165 | 95.7 ± 0.3 | -10.4 ± 0.1 | -78.7 ± 0.3 | -0.3 ± 0.2 | 0.4 ± 0.0 | 6.3 ± 0.4 |
| IL-1R1 | ASN216 | 27.4 ± 0.4 | -6.7 ± 0.6 | -82.2 ± 0.6 | -9.6 ± 0.5 | 0.4 ± 0.1 | -70.7 ± 0.4 |
| IL-1RAcP | ILE135 | 80.4 ± 0.5 | -7.5 ± 0.1 | -58.8 ± 0.2 | -4.4 ± 0.4 | 0.0 ± 0.1 | 10.0 ± 0.8 |
| IL-1RAcP | ASN168 | 20.6 ± 0.5 | -8.5 ± 0.3 | -84.0 ± 1.0 | 2.8 ± 0.8 | 0.0 ± 0.0 | -69.0 ± 0.8 |
| IL-1RAcP | LEU180 | 75.9 ± 1.1 | -9.2 ± 0.3 | -71.2 ± 0.6 | -2.1 ± 0.3 | 0.0 ± 0.0 | -6.6 ± 0.3 |
| IL-1RAcP | ILE181 | 84.4 ± 1.1 | -9.1 ± 0.1 | -63.2 ± 1.3 | -2.2 ± 0.5 | 0.3 ± 0.0 | 9.9 ± 0.2 |
| IL-1RAcP | PRO245 | 80.1 ± 1.1 | -6.1 ± 1.2 | -57.1 ± 2.1 | -1.8 ± 0.9 | 0.5 ± 0.2 | 15.4 ± 2.7 |
| IL-1RAcP | ARG286 | -185.2 ± 0.3 | -9.4 ± 0.4 | 35.8 ± 9.0 | -12.6 ± 8.3 | 0.1 ± 0.0 | -170.9 ± 1.1 |
